# CFD model to study PM$_{\textrm{10}}$ dispersion in large-scale open spaces

**DOI:** 10.1038/s41598-023-33144-9

**Published:** 2023-04-12

**Authors:** V. M. Fernández-Pacheco, E. Álvarez-Álvarez, E. Blanco-Marigorta, T. Ackermann

**Affiliations:** 1 Energy Department, University of Oviedo, Wifredo Ricart s/n, 33204 Gijón, Asturias Spain; 2 Fachhockschule Bielefeld, University of Applied Sciences, Artilleriestr 9, 32427 Minden, Detmold Germany

**Keywords:** Climate sciences, Engineering

## Abstract

Air pollution has become a major concern in industrial or highly populated areas. Although legislation has been enacted to limit pollution levels, air quality monitoring still needs to be carried out by stations which are located at fixed points unable to provide the spatial evolution of pollutants. This research, focused on the city of Gijón (Asturias), includes a Computational Fluid Dynamics model capable of simulating the dispersion of pollutants in a large urban environment (12x18 km$^{\textrm{2}}$). Different wind conditions were simulated with two sources of emission. The results show the influence of the terrain on the dispersion of pollutants in open spaces whilst simultaneously scrutinizing the origin of diffuse industrial pollution circulating over the city of Gijon. The simulation allows us to set limits in the areas with higher levels of contamination or to analyse the variations of particle concentration in height. Therefore, this research defines and validates a methodology to generate numerical models which grant us the opportunity to observe the spatial evolution of pollutants in large areas. This result endorses further use in other lines of research, such as the evaluation of corrective measures to improve air quality in highly polluted environments.

Air quality is one of the major problems in industrial and highly populated areas. Air pollution creates fine particles that cause strokes, heart disease, lung cancer, as well as acute and chronic respiratory diseases. In such a context, the analysis of air quality is of particular importance in order to use the data generated to design measures to reduce air pollution in urban areas, something which is of paramount concern for regional, national and European governments and institutions.

Legislation has been recently introduced to provide a cleaner atmosphere free of anthropogenic pollutants^1^. It is to this end that air quality limit values have been established for some of the main pollutants including those set by the World Health Organisation^2^ and currently enforced through many local regulations. This control is usually carried out by measuring stations located at fixed points^3^. However, such a solution does not allow us to know values between stations, nor to study how the different pollutants (PM$_{\textrm{10}}$, CO$_{\textrm{2}}$, NO$_{\textrm{X}}$...) behave in different scenarios (geometry and meteorological conditions).

Dispersion modelling is one of the most comprehensive and lasting solutions aimed to promote our understanding of the behaviour of pollutants in the atmosphere^4^. Among other solutions, using remote sensing data to create iso-concentration maps with a geostatistical approach, the use of dispersion models such as those recommended by the US-EPA^5^ (AERMOD and CALPUFF) and Computational Fluid Dynamics (CFD) software are the most used tools to performed air quality studies.

There are numerous studies linking statistics and pollutant dispersion. This work is based on using remote sensing data together with ground station measurements to obtain spatially continuous high resolution images. Using the MODIS satellite^6^ and 15 ground stations, images with a spatial resolution of 500 metres can be obtained and may even be enhanced using height or humidity corrections in the boundary layer, thereby improving the results. This satellite works well even in alpine regions and complex terrain^7^. Works such as Roy et al, 2017^8^ estimate PM$_{\textrm{10}}$ concentrations using Landsat 7 in Vadodara (India) at a spatial resolution of 30 metres. The main disadvantage of a geostatistical approach comes from the limited availability of data as it will not be possible to obtain images at every time of the day for the study area.

Most large-scale (macroscale) studies are based on the use of the AERMOD and CALPUFF software tools recommended by the US-EPA. AERMOD has been used to study the spatial variations of SO$_{\textrm{2}}$, NO$_{\textrm{2}}$ and PM$_{\textrm{10}}$ in Pasir Gudag (Malaysia)^9^ using WRF as the meteorological model over a 17x5 km$^{\textrm{2}}$ area with a 100-metre grid. The dispersion results are adjusted because WRF is not able to correctly predict the wind direction in the simulated domain. Another study using AERMOD to predict fine particles (PM$_{\mathrm{2.5}}$) is Michanowicz et al, 2016^10^. The area covered is even larger, stretching 500 km$^{\textrm{2}}$ in Pittsburgh (United States) and considers both short-term meteorological variation and land use. Validation is undertaken at 37 points, all providing satisfactory results. Using CALPUFF, specific cases are also studied. The possibility of similar dry and wet deposition over areas up to 1,000 km is evaluated for a specific case of 137Cs radio nuclides released in 1986 during the Chernobyl nuclear accident^11^. CALPUFF is also used to estimate the impact of the monsoon season on the dispersion of CO, CO$_{\textrm{2}}$ and NO$_{\textrm{X}}$ emitted by vehicles in Salalah (Oman)^12^. Several papers compare the overall results and how they affect each other. In Tartakovsky et al, 2016^13^ the results of AERMOD and CALPUFF are compared with PM$_{\textrm{10}}$ field data from stone quarries in mountainous regions of Israel. AERMOD and CALPUFF both provide a reasonable forecast of pollutant dispersion on a large scale, with low resolution and feasible computational times.

CFD-based techniques are used to obtain highly detailed simulations so that the behaviour of air masses with pollutants can be observed on a micro scale. Existing work focuses on working on small scales, in the order of metres, on streets or blocks that are discretised in detail. CFD has been used to study PM$_{\textrm{10}}$ dispersion in geometries of 1.2x0.5x0.5 m$^{\textrm{3}}$ corresponding to a domain of 34 million cells that attempt to represent a wind tunnel model^14^. In the experiment, a particle emitter and a meter between 1 and 10 microns were used, between which an obstacle was placed. On a slightly larger scale, such as streets of 180x18x18 m$^{\textrm{3}}$^15^ experiments were conducted. Then, this domain was scaled down (1:150) to introduce in the wind tunnel and compare the results of the RANS k-epsilon models with LES, offering a better k-epsilon performance except in the centre of the street where LES enables fluctuations to be monitored but with a huge computational cost.

In larger domains, CIEMAT has used CFD to estimate the spatial representativeness of NO$_{\textrm{2}}$ from air pollution monitoring sites in two urban districts of Madrid^16^. The locations were “Escuelas Aguirre” with an area of 700x800 m$^{\textrm{2}}$ (3 million 1-3 metre cells) and at a height of 540 metres, including part of the retreat, and Plaza Castilla with an area of 1,000x1,000 m$^{\textrm{2}}$ (2 million 1-3 metre cells) and 570 metres high. The geometry includes buildings and an irregular grid with 1 to 3 metre cells and a height over 500 metres. The results indicate that one station is more representative than another due to differences in urban structure that affect ventilation. CFD models have also been used to evaluate the aerodynamic effects of trees on a local scale, studying the influence of green infrastructure on the dispersion and removal of pollutants from roads in models of 180x18x36 m$^{\textrm{3}}$^17^, 160x40x30 m$^{\textrm{3}}$^18^ and 18x26x180 m$^{\textrm{3}}$^19^. The three studies represent along the same idea based on the construction of a scale model of the street canyon to be tested in a wind tunnel and its modelling in CFD to evaluate the influence of different types of vegetation. Using OpenFOAM^20^, the ability to remove pollutants from a street and its relationship to breathability and airflow exchange rate was investigated by analysing the pollutant exchange rate. The results indicate that the pollutant removal rate is dominated by turbulent pollutant diffusion^21^.

On a much larger scale, there is an experiment by Liu et al, 2018^22^ using a numerical simulation and physical model to analyse the effects of complex building environments on gas leakage. The experimental part involved simulating natural gas leakage and diffusion to determine the impact of area winds, conducting outdoor tests and placing methane concentration sensors to capture and verify concentration changes due to wind variations. This experiment demonstrated that the proposed CFD model could be used to simulate and predict the diffusion of natural gas in the event of an accidental release.

CFD models have also been compared against other models. The study^23^ compares the results of a statistical model (ALOHA 5.4.3) and a CFD model (ANSYS Fluent 13.0) for the study of the dispersion of toxic substances in the atmosphere resulting from accidents. The results of the two simulations were shown to differ radically, with the CFD model providing better quality results when dealing with accidents. This other work^24^ also evaluates the dispersion of pollutants in an urban environment using a CFD model, a Gaussian model and the semi-empirical ASHRAE model by comparing the results with data obtained in a wind tunnel. Numerical simulations with CFD offer some advantages compared to other methods. Firstly, it is said to be less costly than wind tunnels and measurement campaigns. Moreover, the results are provided at every point of the domain simultaneously. However, CFD requires great caution in its setup so that the results may be reliable.

In this research, an area in Gijón (Spain) has been selected as one of the worst locations according to the number of times daily limit values for PM$_{\textrm{10}}$^25^ were surpassed. A CFD numerical model is assembled which considers the overall terrain, creating a 3D computational territory able to simulate the dispersion of pollutants in open spaces on a large scale (12x18 km$^{\textrm{2}}$). The CFD model, which was previously validated by wind tunnel tests, makes it possible to reproduce meteorological conditions and to work in large industrial areas in order to obtain 3D analysis of industrial pollutants.

## CFD methodology

### Study area

The first step of a CFD model is to select the study area. In this case, the western area of Gijón has been selected because it is one of the places in Spain at which the highest number of daily limit values of PM$_{\textrm{10}}$ have been surpassed according to the Ministry of Ecological Transition^25^. More specifically, an area of 18,000x12,000 m$^{\textrm{2}}$ was selected, covering the industrial zone of Gijón, the urban centre and the surrounding mountains. In addition to its extreme degree of pollution, this area stands out for its uniqueness due to the proximity between the sea and the mountains.

**Figure 1:**
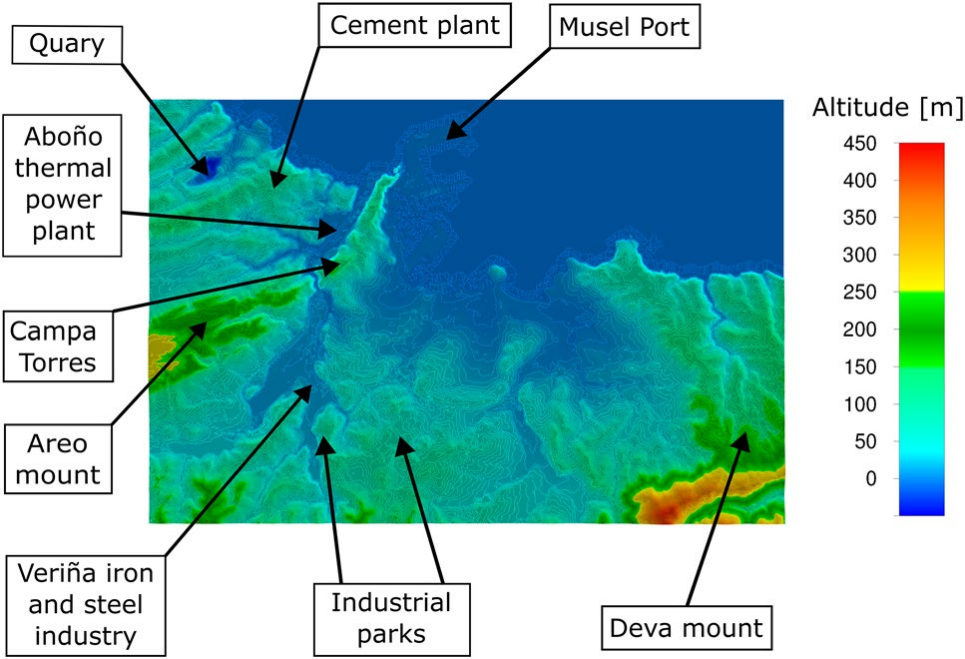
Areas of interest and altimetry of the study area (ANSYS Fluent 19.2 (2019)).

The territory chosen for simulation includes the Cantabrian Sea coast to the north, Campa Torres and Monte Areo (west) and Monte Deva (southeast). As for the industrial areas, the most important ones are those in the Veriña iron and steel area, the Aboño thermal power plant, the Musel Port, as well as a cement works, a quarry and numerous industrial parks. The elevation varies from sea level to a maximum of 426 m in the mountainous area (Fig. 1).

### Geometrical model

Once the study area has been defined, a geometric model of the area is assembled. The surface of the study area measures 12,000 x 18,000 m$^{\textrm{2}}$ and has been acquired from IGN (National Geographic Institute) data. It is a free model created through LIDAR flights of the National Aerial Orthophotography Plan (PNOA) and has been processed to obtain a spatial resolution of 5 meters (MDT05). These files are obtained in ASC format for a region of Spain. The next step is to crop the DTM to the required terrain coordinates in order to adjust the territorial boundaries.

Before meshing the study area, pre-processing is undertaken to simplify the surface and transform it into a STereoLithography (STL) format file which will then be imported by the meshing software. The structure of the study area will be formed by this artificial terrain and a flat upper surface located at an altitude of 3,000 m, which will correspond to the ceiling of the simulated atmosphere.

**Figure 2:**
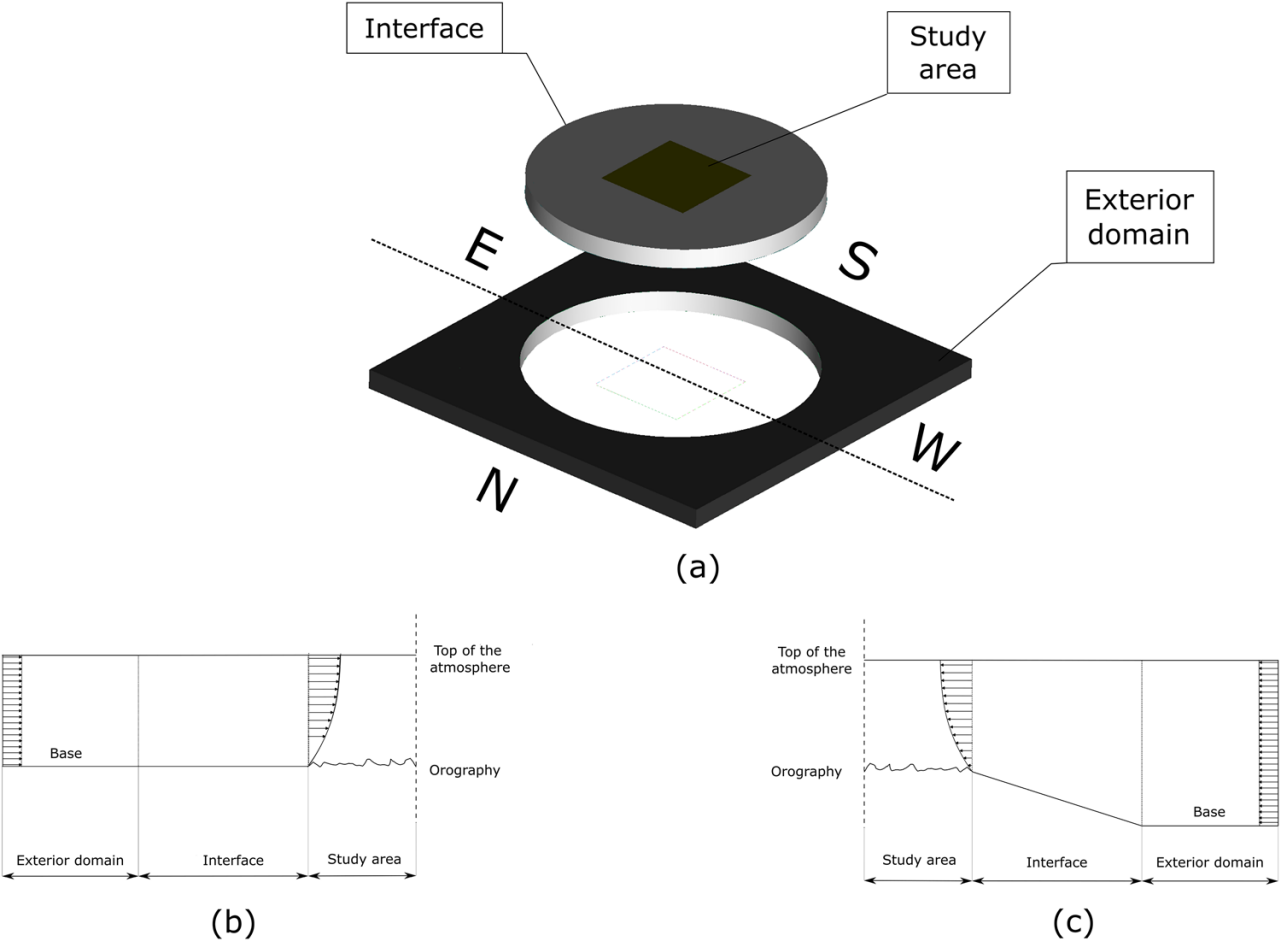
(**a**) Complete geometric model and meshed areas. (**b**) Cross section in coastal area. (**c**) Cross section in mountainous area.

The CFD model represents a virtual wind tunnel with three zones (Fig. 2a): (1) the study zone in the centre, (2) an interface attached to it, (3) an exterior domain. The circular shape of the interface zone allows both zones to rotate with regard to the external domain and create the necessary angle to simulate different wind directions without geometrical modifications. Wind speed is included as a uniform profile on one side of the domain, develops as it passes through the exterior domain and the interface zone until it is fully developed when it reaches the zone under scrutiny. An additional function of the interface zone is to adapt the elevation of the base of the exterior domain plane to match the ground surface of the study area with the use of a slight slope, so that all points on the outside of the domain have an elevation of 0. Figure 2b shows the profile of the coastal zone and Fig. 2c shows the profile of the mountain zone.

### Numerical model

Once the geometry has been developed, the next step is to mesh the whole volume. In the study area, tetrahedral cells with a size of 60 m are used for the first 500 m in order to capture the irregularities of the terrain. From 500 m to the top of atmosphere (3,000 m), triangular-based prisms are used, the spacing of which increases up to 250 m. In the interface zone and in the exterior box, triangular-based prisms are used, with horizontal spacing reaching 1,000 m and vertical spacing 250 m. In total, a grid of 2,855,455 cells is obtained.

Mesh quality analysis yielded very satisfactory results, indicating an equitable skew value below 0.4 for 100 % of the mesh cells. This parameter shows the shape of the cells between 0 and 1; values close to 0 indicate more regular cells, which are much more likely to achieve satisfactory results. Tests were performed with a large number of cells (5 million) without appreciable variations in the results.

In order to model the conveyance of the pollutant within the air mass, a multiphase model is used, of which there are different models (discrete phase model, Eulerian model, mixing model, volume-fluid model) due to the great diversity of existing fluids and regimes. Each model has been developed to suit a specific situation, the Eulerian model being the one used in this case. The Eulerian multiphase model, which is the most sophisticated and general model can be used to model multiple phases, which may be in liquid, gaseous or solid state form. The model uses an Eulerian approach for all phases and focuses on solving control volumes. The model solves the continuity, quantity of motion and energy equations for each phase, but the pressure field is unique for all phases.

The volume fraction is defined for each q-th phase, denoted as $\alpha _q$ as the space occupied by each phase (in terms of one), satisfying:

1 $$\begin{aligned} V_q=\int _{V}\alpha _q\cdot dV \qquad \sum _{q=1}^{N}\alpha _q=1 \end{aligned}$$

Thus, for each phase we define a continuity equation and the three equations of quantity of motion.

### Boundary conditions

**Figure 3:**
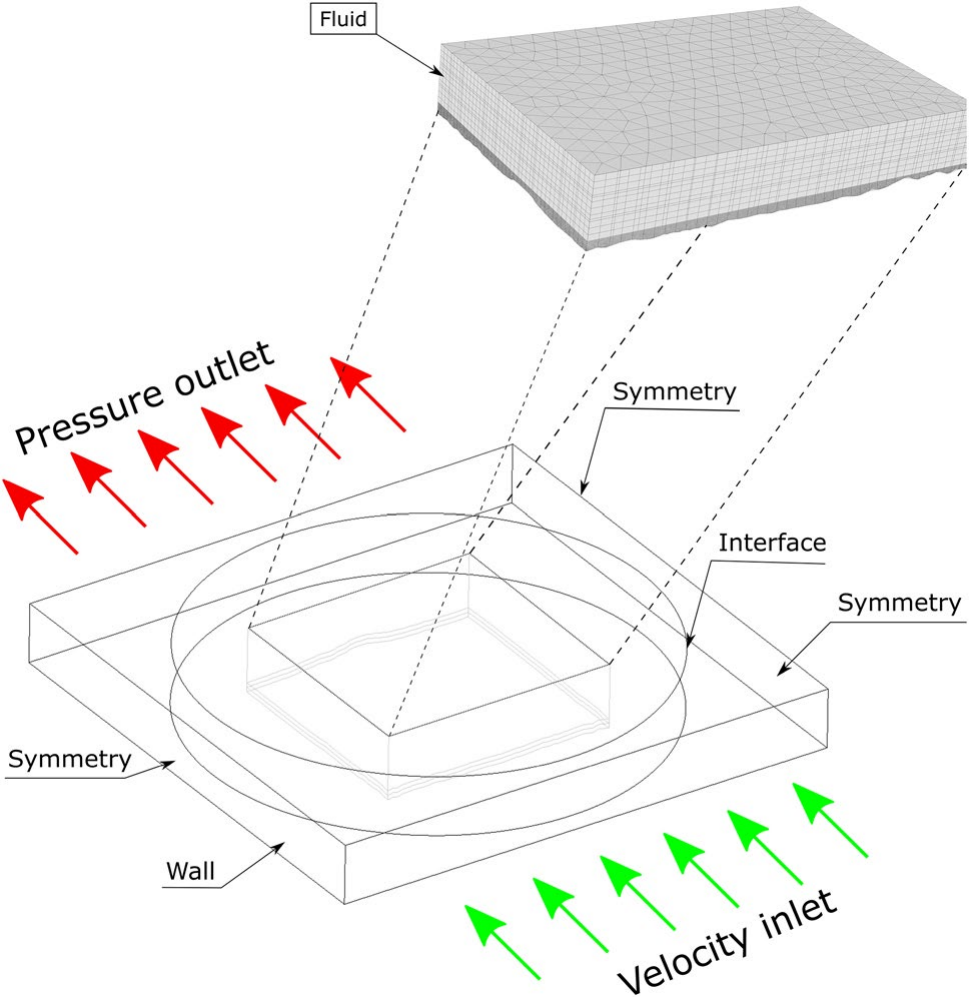
Boundary conditions.

Different boundary conditions have been considered to simulate different processes affecting the evolution of the meteorological variables in the same model. In the external domain, conditions are considered to recreate an environment including meteorological variables (Fig. 3). An initial surface is set as a velocity inlet with a view to introducing a uniform profile of wind speed, humidity, and temperature. The opposite surface is set as a pressure outlet. The other two side faces are set as a frictionless wall or symmetry and the same for the top of the atmosphere’s surface. The bottom surface is defined as a rough wall. An interface condition will allow the cylinder to rotate with respect to the domain, offering the possibility to select a precise angle for the wind inlet.

**Figure 4:**
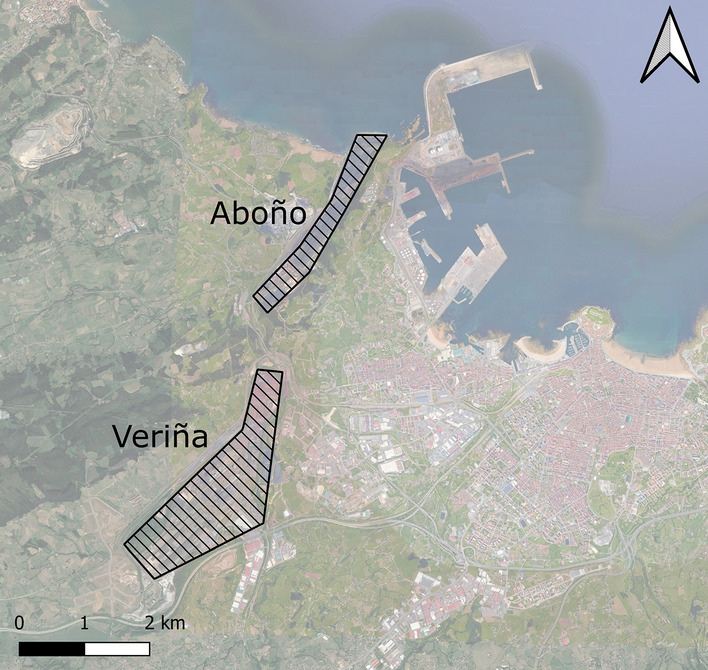
Location of emitting sources (QGis 3.28).

In addition, a set of boundary conditions is applied to the four boundary surfaces in the area under investigation. The upper one, known as “top of the atmosphere”, is defined as a frictionless wall. The intermediate boundary is defined as an interface, the land surface is a rough wall. Finally, the lateral surfaces of the study area are all interfaces. Two areas corresponding to the industrial area of Aboño and Veriña have been defined. In these areas, two velocity outlets have been located in which a very low velocity inlet (in the order of 0.05 m/s) and a concentration of PM$_{\textrm{10}}$ particles as a volume fraction have been defined. These zones can be seen in Fig. 4.

### Input data

In general terms, according to the West Gijón Air Plan^26^, the pollutant sources around the city of Gijón that affect its pollution levels represents diffuse pollution due to the Aboño thermal plant in the west, the Veriña industrial area and the industrial parks in the southwest and traffic on the A8 motorway to the south.

**Figure 5:**
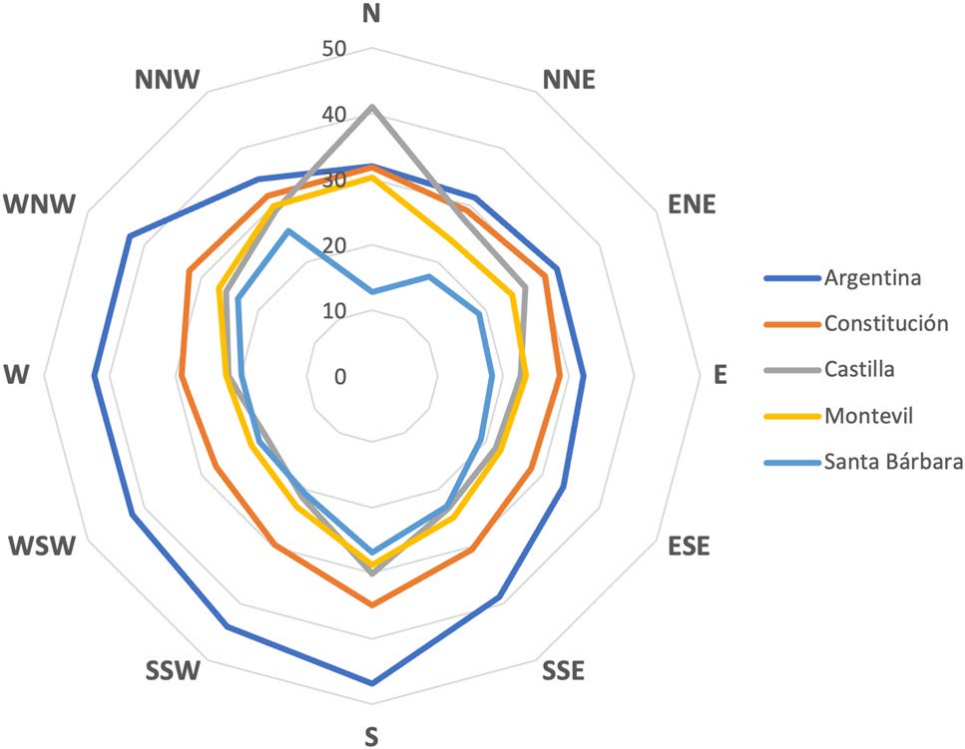
Annual mean PM$_{\textrm{10 }}$ ($\mu$g/m$^{\textrm{3}}$ ) per station for each wind direction.

Wind data are downloaded every minute from the meteorological station located at the Port of Gijón (5.70$^{\textrm{o}}$ W; 43.56$^{\textrm{o}}$ N)^27^. These data are cross checked/contrasted with the data from the air quality stations [3] to obtain the mean PM$_{\textrm{10}}$ distribution for each station as a function of wind angle, building the diagram in Fig. 5.

**Figure 6:**
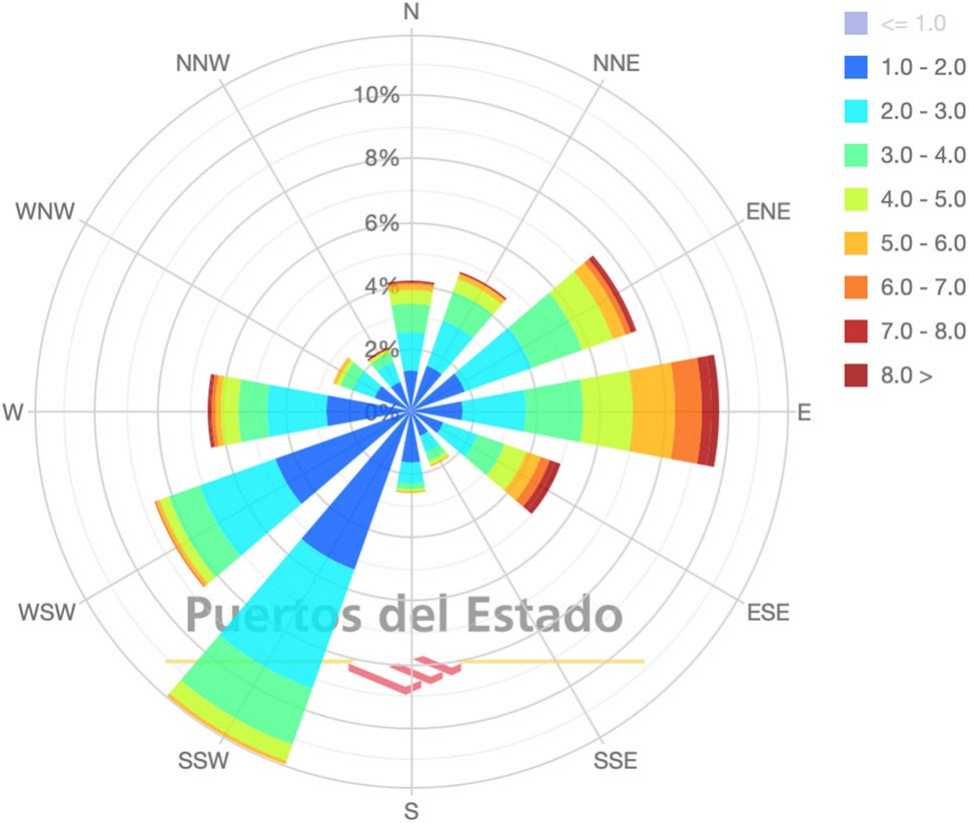
Wind rose for the Port of Gijón for average speed (m/s) in the period 2012-2022. Source: Puertos del Estado^27^.

It can be seen in the PM$_{\textrm{10}}$ diagram of particulate matter how the highest values reached in the city of Gijón correspond to the station located in Avenida de Argentina (La Calzada neighbourhood), one of the most polluted neighbourhoods in the city. Specifically, the highest values occur when the winds have a south-westerly component due to the joint effect of both sources (Veriña and Aboño). Above-average values are also observed in the south, but these may be due to pollution from road traffic around the city and industrial parks on the outskirts. Winds from the south carry particulate matter towards the centre and increase the concentration at all measuring stations. The wind rose diagram for the city of Gijón for a 10-year period (2012-2022) is also obtained from the Port meteorological station (Fig. 6).

All wind directions were simulated, using at each angle the average and maximum wind value for the year 2021 and for the surfaces corresponding to the emitting source, a concentration in outlet volume of 10$^{\mathrm{-7}}$ PM$_{\textrm{10}}$ was considered (secondary phase of the multiphase model with density 2000 kg/m$^{\textrm{3}}$, the primary phase being air) with an outlet velocity of 0.05 m/s and an average particle size of 10 microns. With these data, in the cases of wind with a westerly component, the pollution of industrial origin at the station located on Avenida de Argentina will be intermediate between the values assigned to these industrial sources by the studies that said between 40 % in the Gijón Oeste Plan^26^ and 60 % in other works^28^ that had a great impact on the media^29^. Therefore, the aim is to ensure that the values arriving at the stations are within the same order of magnitude, but with lower values than those measured, as there will still be other industrial sources, traffic, diffuse pollution, etc. that contribute to increasing particle emissions.

### Simulation conditions

Numerical calculations were performed with Ansys Fluent 19.2 (2019) software. The SIMPLE (Semi-Implicit Method for Pressure-Linked Equations) algorithm was used to solve the coupling between the pressure and velocity fields. A second order spatial and temporal discretisation scheme was employed, PRESTO! was used as the pressure scheme and the k-Epsilon-RNG model was used as the turbulence model.

In an attempt to replicate real conditions, it is necessary to carry out two simulations. An initial steady-state simulation that allows the wind to develop and the model to warm up. For this simulation, only the velocity inlet condition corresponding to the wind speed and the atmospheric pressure condition is placed at its end. Once this solution has converged, a transient simulation is carried out where the effect of the source is considered. This second part involves the largest computational effort, as it also includes the Eulerian-type multiphase model with its two phases: (1) air for the main component and (2) PM$_{\textrm{10}}$ particulate matter 10$^{\mathrm{-7}}$ for the emitting sources.

Twelve simulations were performed for each wind speed, one for each wind angle (at 30$^{\textrm{o}}$ intervals) and for two different wind speeds (mean and maximum wind). For the transient part, 200 time intervals of 60 s each were simulated with enough iteration to reach values below 10$^{\mathrm{-5}}$ for the normalised residuals of each governing equation. In total there are 24 simulations with a computation time of approximately 20 hours on a computer with a 16-core AMD EPYC 7351P processor at 2.4 GHz running Ansys Fluent 19.2 (2019).

## Model validation

**Figure 7:**
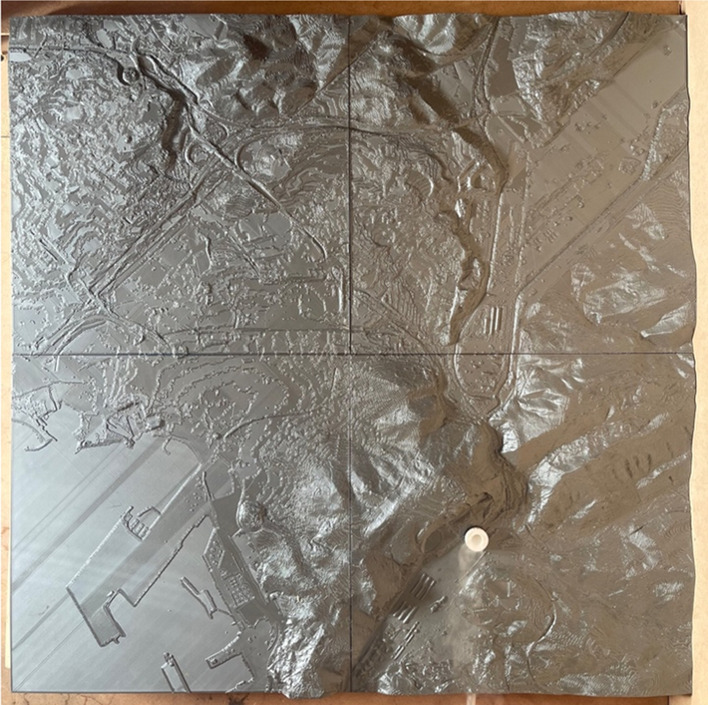
3D printed scale model used for validation (photo taken by the authors).

Validation of the model is carried out by wind tunnel tests where it is placed on a reduced scale physical model and its results are compared with those on a small-scale CFD model. For this purpose, a small-scale physical model made by 3D printing and a particle emitter manufactured for the test were used. Measurements were taken for the four predominant directions using a particle sizer (Fig. 7).

**Figure 8:**
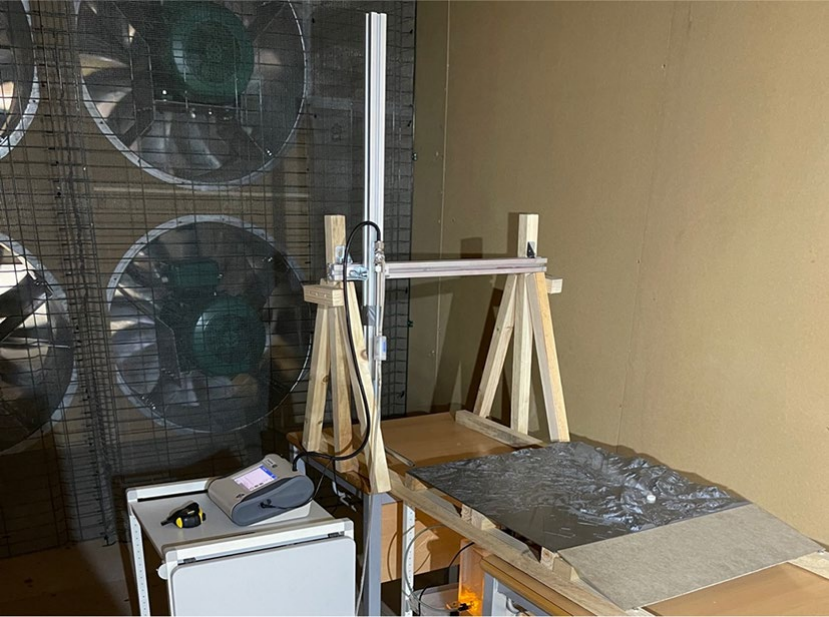
View of the physical model tested in the wind tunnel.

Therefore, to carry out the validation a CFD numerical model at a reduced scale will have to be built. For this purpose, the model described above is taken as a foundation and a reduction scale of 1:10,000 is considered in all axes. The chimney or source of pollution changes with respect to the real values. In the laboratory model, a single emitting source was used with a height of 70 mm above the 0 level of the model and a diameter of 20 mm, with an outlet of 10 mm. This emitting source is located in the Aboño area of the large-scale model (Fig. 8).

The input data for the model are the wind speed (m/s) selected in the wind tunnel and the characterisation of the pollutant source. Concentration and velocity (m/s) of the chimney outlet were determined. Thus, in accordance with laboratory data, a wind speed of 0.4 m/s perpendicular to the model and uniformly distributed was considered for these simulations. For the stack, an outlet volume concentration of 1 % liquid water (secondary phase of the multiphase model, the primary phase being air) with an outlet velocity of 0.05 m/s and an average particle size of 5 microns was considered.

**Figure 9:**
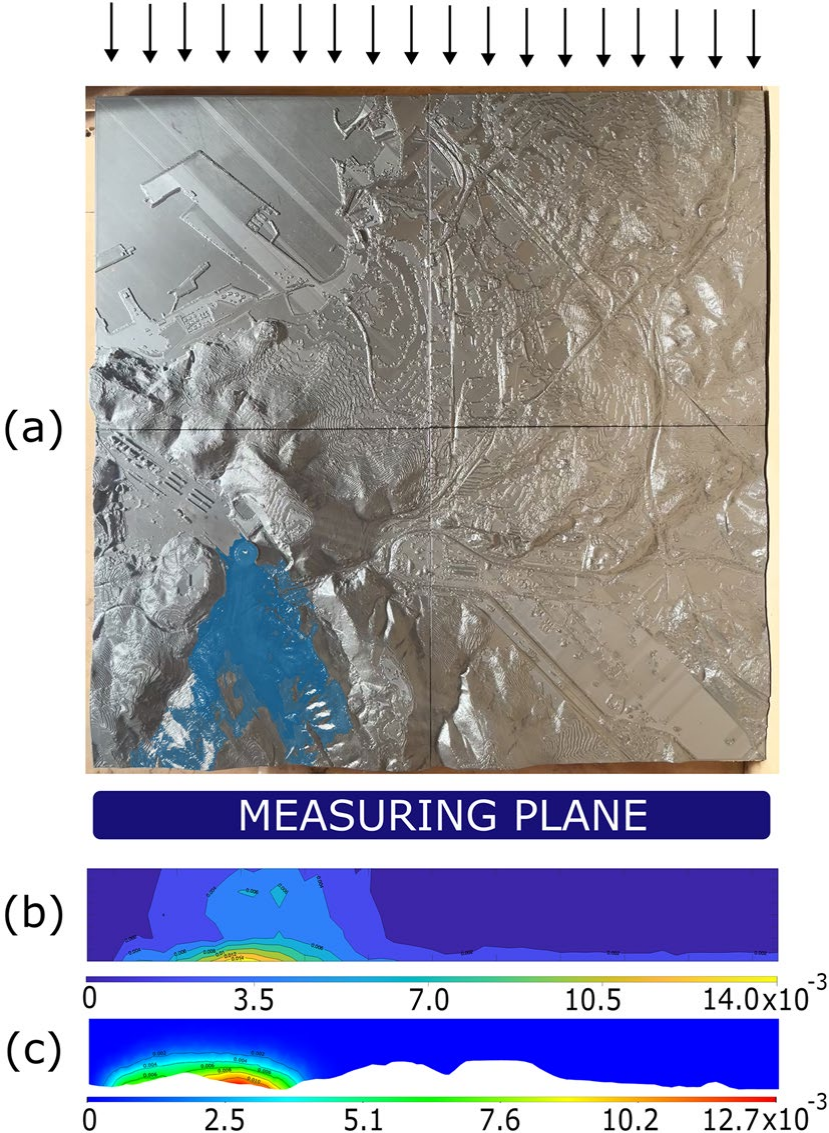
(**a**) View of the experimental model with east wind (photo taken by the authors). Comparison between (**b**) experimental data and (**c**) CFD simulation data.

The results provided by the CFD simulations at a reduced scale are similar to those obtained under laboratory conditions. The model shows the ability to predict the behaviour of particle dispersion under different wind conditions and taking into consideration the influence of the terrain employed in the model. The values have remained in the same order of magnitude, with a tendency of the CFD to underestimate the maximum compared to the experimental data. In addition, values of the sample correlation coefficient (https://en.wikipedia.org/wiki/Correlation#Sample_correlation_coefficient) are above 0.8 in all cases. Figure 9(a) illustrates a view of the experimental model with easterly wind. Below, a comparison between experimentally measured data (b) and CFD simulation data (c) is shown.

## Results and discussion

**Figure 10:**
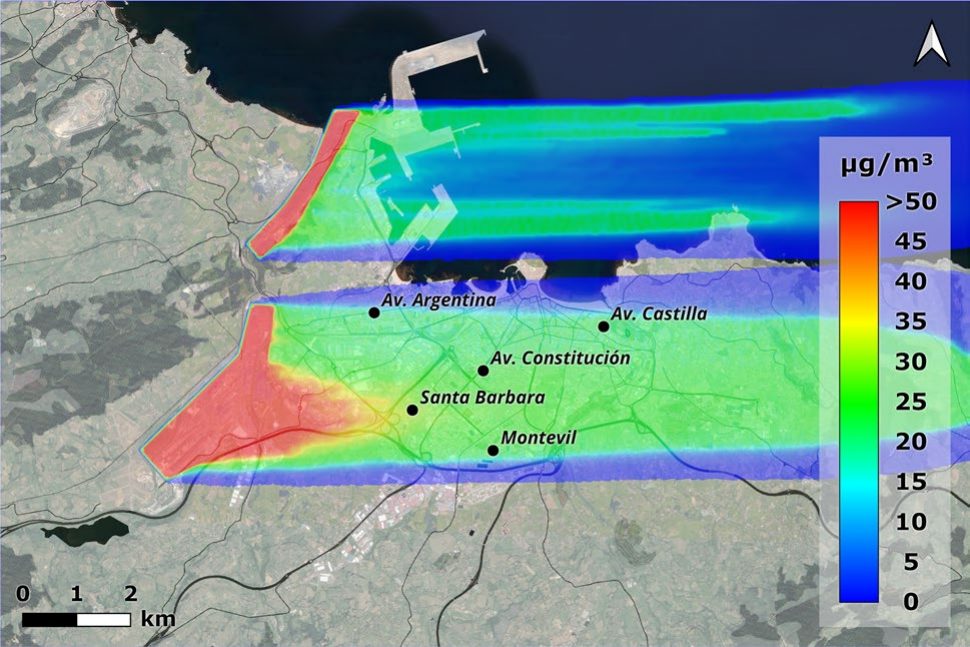
Concentrations obtained with an incident wind from the west (270$^{\textrm{o}}$) at annual average speed of 1.54 m/s (ANSYS Fluent 19.2 (2019) & QGis 3.28).

Once the numerical model had been validated through experimental tests in the wind tunnel, full-scale simulations were carried out with the CFD model. The results of these simulations provide insight into the influence of the terrain, wind direction and wind speed on the dispersion of pollutants in large-scale open spaces in 3D. This paper shows the results for the two prevailing winds: east (90$^{\textrm{o}}$) and south-southwest (210$^{\textrm{o}}$) and, additionally, west wind (270$^{\textrm{o}}$) because it is unfavorable for pollution in Gijón. In the case of the westerly wind (Fig. 10) it can be seen how the wind carries pollution directly towards the city, with values of up to 30 $\mu$g/m$^{\textrm{3}}$ of PM$_{\textrm{10}}$ in the central area, being even higher in the western area. The Campa Torres acts as an initial barrier for the Aboño source, but the particles continue to pass through it as the wind blows from the west. In the case of the Veriña source, particles are clearly dragged towards the city, reaching very high values around the Santa Bárbara station.

**Figure 11:**
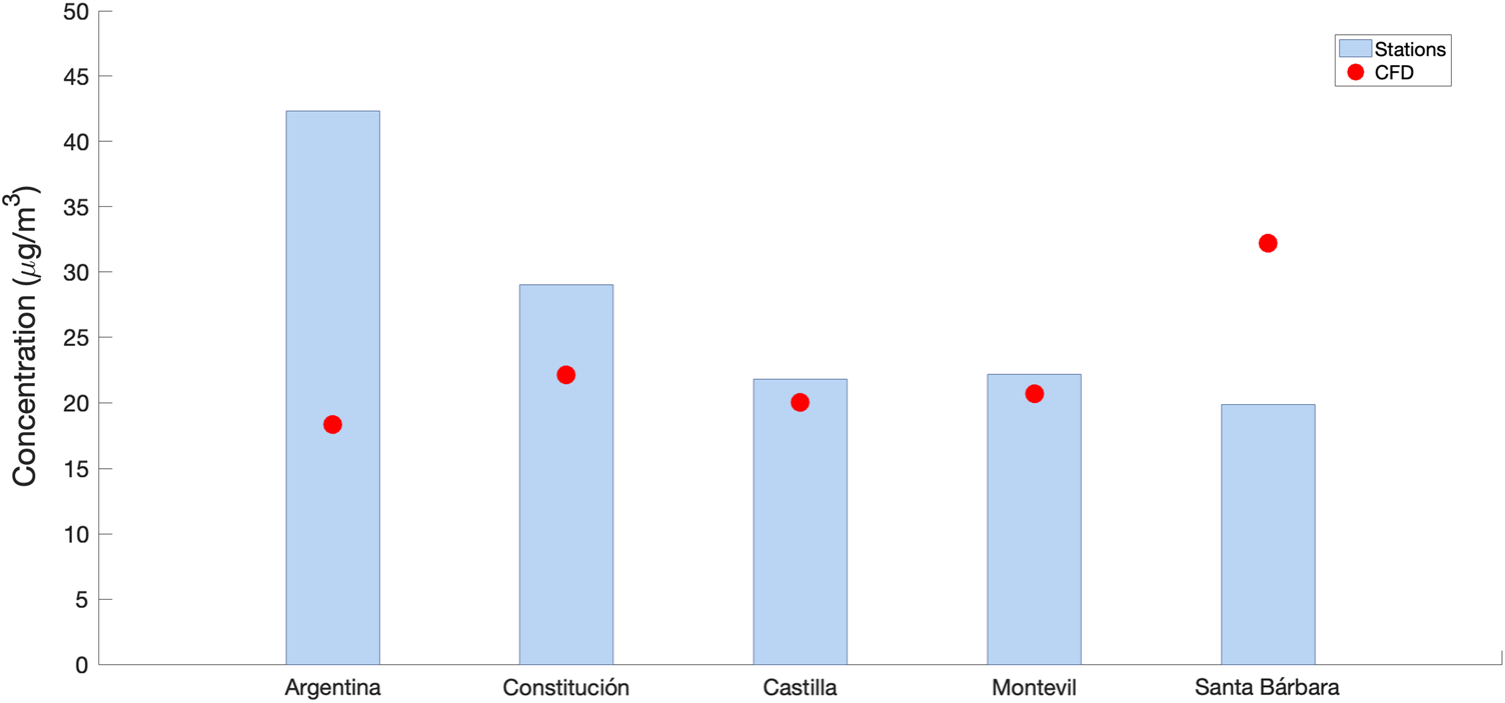
Comparison between measuring stations and CFD values for a 1.54 m/s westerly wind (annual average speed).

Figure 11 shows the comparison between the simulation results and the measured annual mean values. It represents the CFD values, which are in most cases below what was initially predicted. In the case of Avenida de Argentina, the CFD results are around 50 % of those measured at the stations, with the westerly wind directly bringing the pollution from the simulated industrial sources. Thus, we have values with percentages of industrial pollution similar to those collected by the aforementioned studies. The excess at the Santa Bárbara station is due to the fact that, for this area, the influence of the westerly wind is critical, bringing the particles from the Veriña source directly towards the town. While to the west the Campa Torres and Monte Areo help to mitigate the effect of pollution, to the east the terrain is practically flat, which means that in episodes of westerly wind there is no natural barrier to prevent particles from reaching the area. However, the actual measurements of Santa Bárbara station are mitigated by its incorrect location (43.522912, -5.689480), as it is behind a vegetation barrier, which in westerly winds diminishes the results.

**Figure 12:**
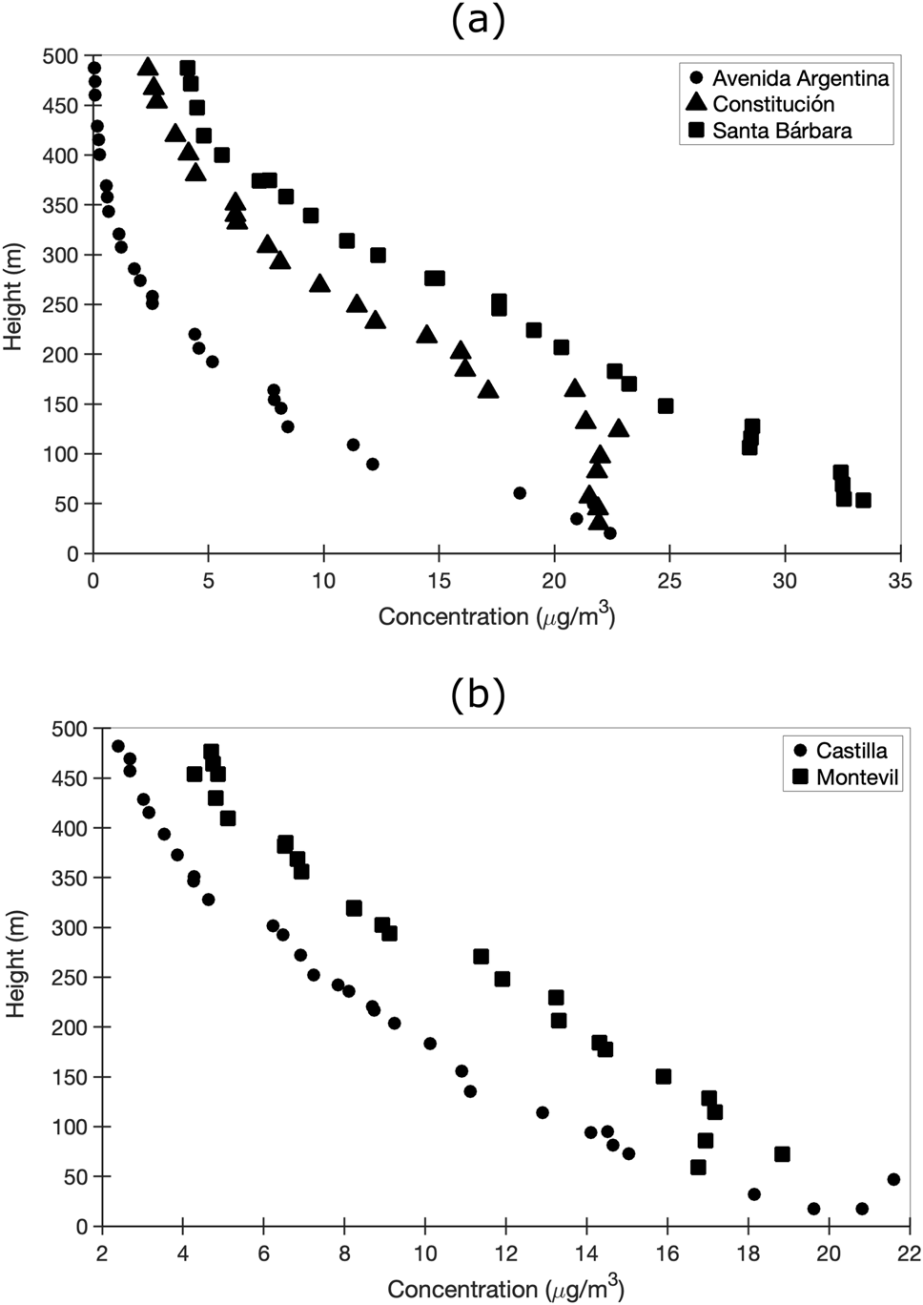
Concentration profiles at height for air quality stations located in Gijón with an incident wind from the west (270$^{\textrm{o}}$) at a speed of 1.54 m/s (annual average speed). (**a**) Avenida Argentina, Constitución and Santa Bárbara; (**b**) Castilla and Montevil.

Figure 12 shows the PM$_{\textrm{10}}$ concentration profiles in $\mu$g/m$^{\textrm{3}}$ high for the westerly wind simulation (270$^{\textrm{o}}$) in five locations: (a) Avenida Argentina, Constitución and Santa Barbara, (b) Castilla and Montevil. The first value corresponds to the lowest elevation, which is equivalent to the measurements of the air quality station located on the ground. For the remaining values, it can be seen how the particles are dispersed in the first 500 m of the simulated atmosphere.

Crosswinds when the wind is favourable allow us to ascertain the full influence of the terrain. In the simulation, under unnatural and invoked conditions, two large pollution sources have been located (of the order of 100 hectares in Aboño and 300 hectares in Arcelor) from which particles are released at very low velocity at ground level. Therefore, the terrain is of great importance, as it is capable of directing pollution towards different areas.

**Figure 13:**
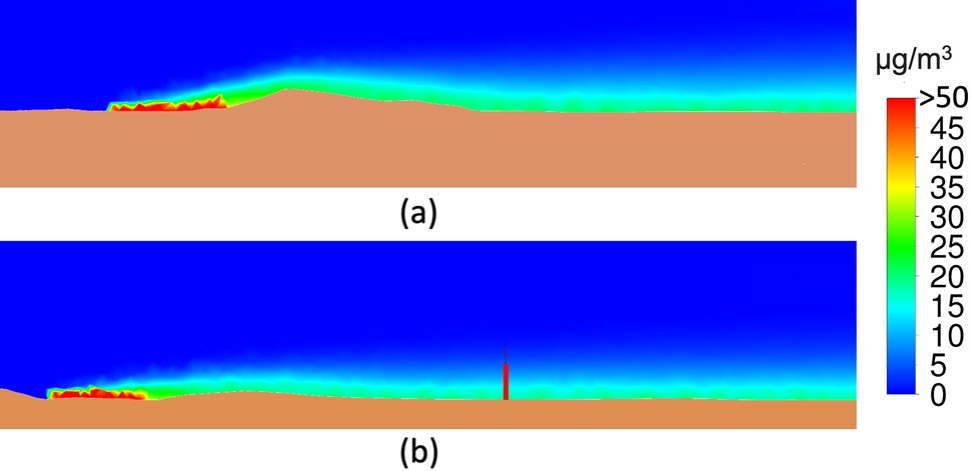
Planes in the direction of the incident wind: (**a**) At the height of Aboño including Campa Torres. (**b**) Passing the air quality station at Avenida de Argentina (red).

Figure 13a shows the plane passing through Campa Torres and its effect as a barrier to the dispersion of pollutants. Figure 13b shows the onset of pollution from Veriña to the city, passing through the air quality station at Avenida de Argentina (La Calzada neighbourhood), whose location is shown below.

Both planes are from the simulation with a westerly wind (270$^{\textrm{o}}$), where the incidence of a direct wind from the source over almost flat ground causes the particles to disperse throughout the city, which is why in this case there are PM$_{\textrm{10}}$ records at all the air quality stations.

**Figure 14:**
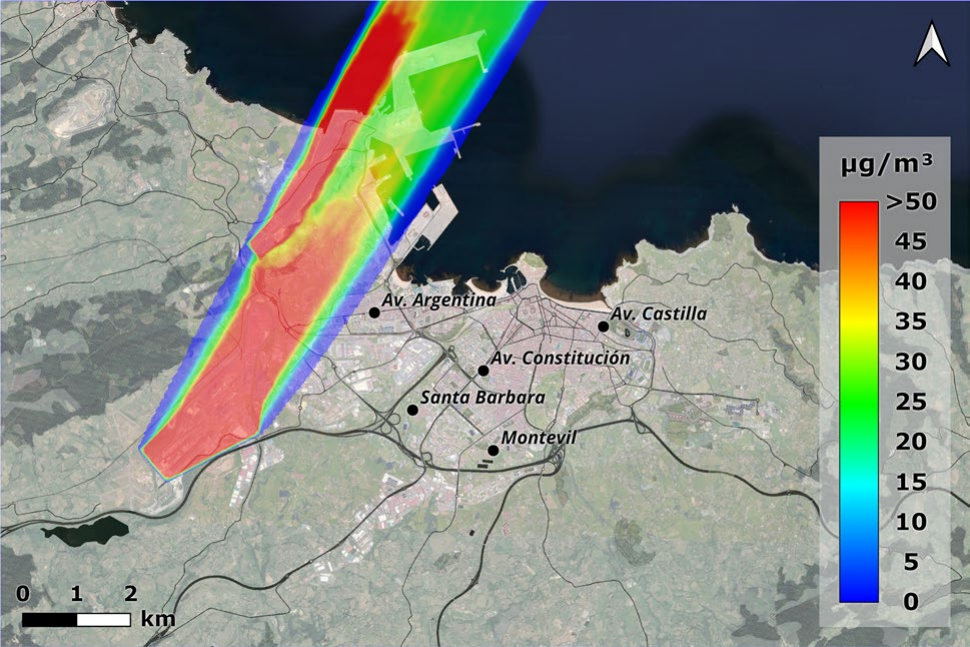
Concentrations obtained with an incident wind from the south-southwest (210$^{\textrm{0}}$) at a wind speed of 1.66 m/s (ANSYS Fluent 19.2 (2019) & QGis 3.28).

Looking at the wind rose diagram, one of the predominant wind directions in Gijón, especially in autumn and winter, is from the south-soutwest (210$^{\textrm{o}}$). With this arrangement and the geometry of the focal points, thetams e pollution plumes are practically aligned. The area west of Gijón would be the most affected, as it receives all the particles from the industrial area of Veriña (Fig. 14). In this case, values above 50 $\mu$g/m$^{\textrm{3}}$ would be obtained within the city, which, according to WHO recommendations, are very high for average values.

**Figure 15:**
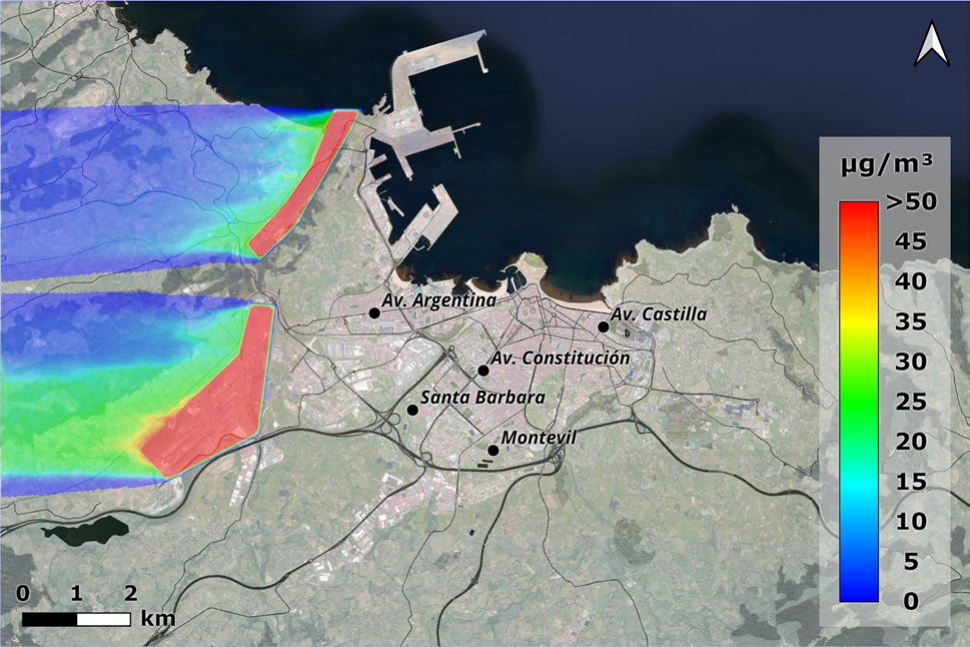
Concentrations obtained with an incident wind from the east (90$^{\textrm{o}}$) at a speed of 2.75 m/s (ANSYS Fluent 19.2 (2019) & QGis 3.28).

Another predominant wind throughout the year is the east wind (90$^{\textrm{o}}$). When simulated everything points to it being a positive wind for the city, since most of the industry is located in the western area, which moves pollution away from the most densely populated area. Figure 15 shows how, despite the proximity to the pollution sources, Monte Areo serves as a natural barrier and prevents pollution from reaching some areas in the west of the territory. The values in the city are zero, as in the simulation there are no other sources that could be present in these areas (mainly due to traffic) and could be influenced by this wind.

What this highlight is that the influence of the industrial areas of Aboño and Veriña is considerable when the wind blows from the west but has a significantly reduced impact to the values of the city stations when the prevailing winds come from the south-southwest and east. In this case, particle pollution (of greater magnitude, outside Gijón) is due to other non-simulated sources of pollution such as the industrial estates in the south-west area together with road traffic.

## Conclusion

The damage caused by air pollution is a major worldwide concern which should not be underestimated, with hundreds of people exposed to polluted air every day. In this research, a CFD numerical model is put forward that takes into account diverse terrain, creating a 3D computational domain capable of simulating the dispersion of pollutants on a large scale. For validation, the original numerical model is scaled up (1:10,000) to include a particle emission source whilst a physical model is built on the same scale using 3D printing. The model is tested in the aerodynamic tunnel in the main wind directions. The results are satisfactory as the numerical model shows a great capacity to predict the behaviour of the dispersion of particles in the air.

The results of the validation phase allow the model to be used on a large scale adopting the city of Gijón as an example. All wind directions were simulated, using two wind speed values (average and maximum).

The input concentration used allows industrial pollution values at the Avenida de Argentina station (in the case of westerly wind) to be between 40 and 60% (percentages assigned to these sources by different studies). In these simulations, the high pollution recorded in the western area can be clearly observed, as a result of the proximity of the industrial sources analysed. The results are in the same order of magnitude as those observed in the station data. The model developed helps us to understand the way terrain affects the behaviour of industrial pollution particles on a large scale in 3D and clearly demonstrates its potential.

These results give rise to future work to study other sources of industrial origin, as well as the study of different scenarios (climate change, pandemics, etc.) which modify the sources of emissions or the analysis and selection of corrective measures to improve the air quality of the population in industrial environments.

**Supplementary information.** Images of the 24 simulation cases can be found in the following link: Suplementary-Material.

## Supplementary Information

Supplementary Information.

## Data Availability

We only used data from public sources. Digital Terrain Model can be downloaded at: http://centrodedescargas.cnig.es/CentroDescargas/catalogo.do?Serie=LIDAR. Wind data can be downloaded at: https://www.puertos.es/es-es/oceanografia/Paginas/portus.aspx. Air pollution data (PM$_{\textrm{10}}$) can be downloaded at: https://asturaire.asturias.es/.
