# Supplementary material for: CFD model to study PM10 dispersion in large-scale open spaces
Source: Sci Rep. 2023 Apr 12;13:5966. doi: 10.1038/s41598-023-33144-9 (PMC10097623; doi:10.1038/s41598-023-33144-9)
Supplement: Supplementary file 1 — Supplementary Information. [file 41598_2023_33144_MOESM1_ESM.pdf]

**SUPPLEMENTARY MATERIAL: COMPLETE LIST OF SIMULATIONS**

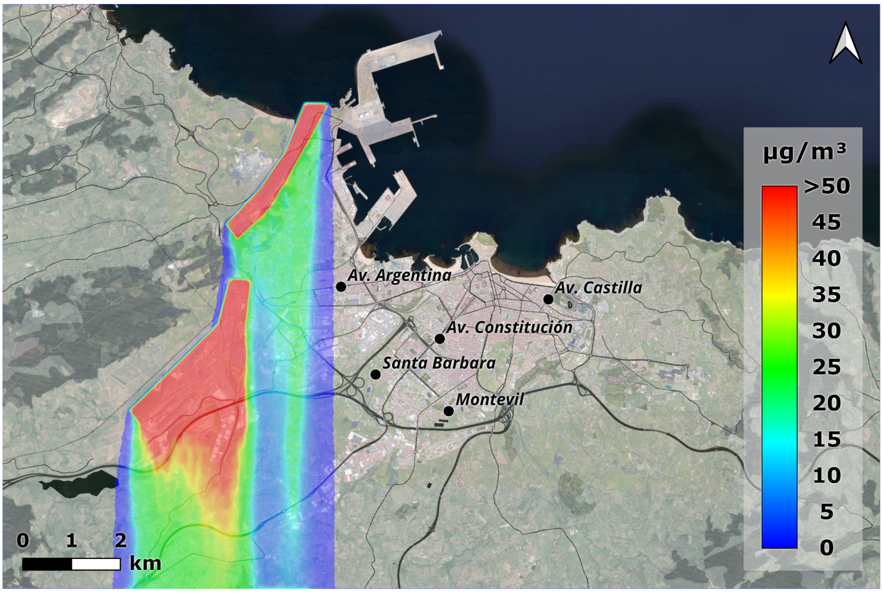


Case 1: $PM_{10}$ dispersion for North wind (0º) at annual average speed of 1.79 m/s (ANSYS Fluent 19.2 (2019) & QGis 3.28).

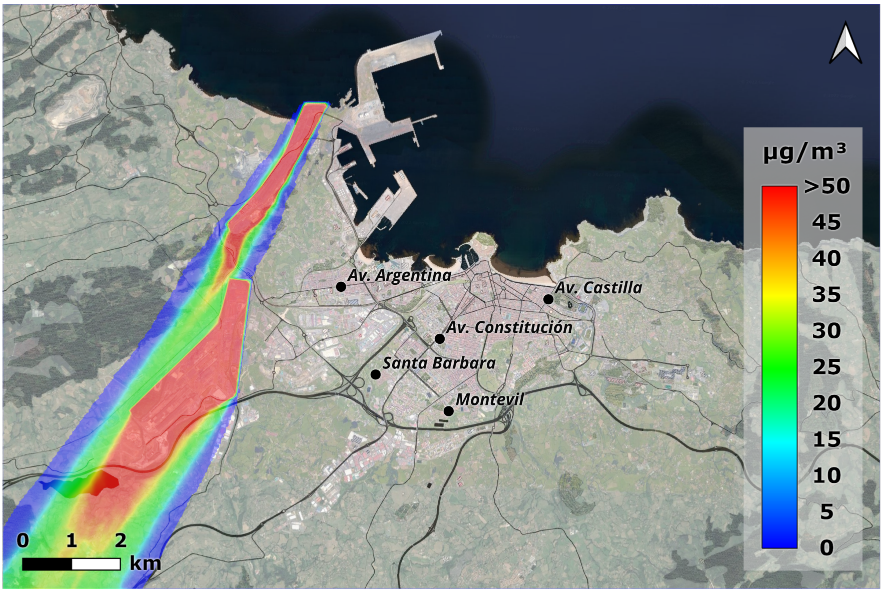


Case 2: $PM_{10}$ dispersion for North-Northeast wind (30º) at annual average speed of 2.00 m/s (ANSYS Fluent 19.2 (2019) & QGis 3.28).

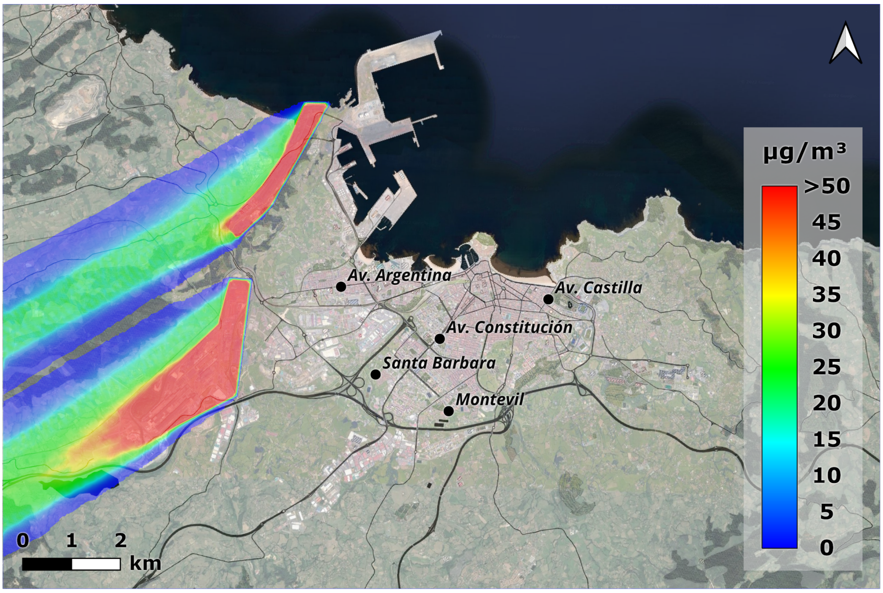


Case 3: $PM_{10}$ dispersion for East-Northeast wind (60º) at annual average speed of 2.21 m/s (ANSYS Fluent 19.2 (2019) & QGis 3.28).

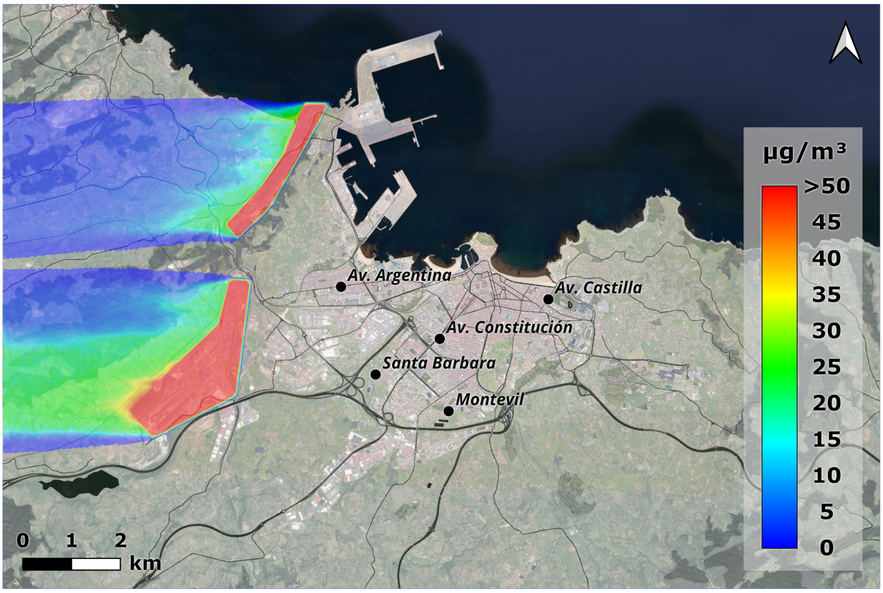


Case 4: $PM_{10}$ dispersion for East wind (90º) at annual average speed of 2.75 m/s (ANSYS Fluent 19.2 (2019) & QGis 3.28).

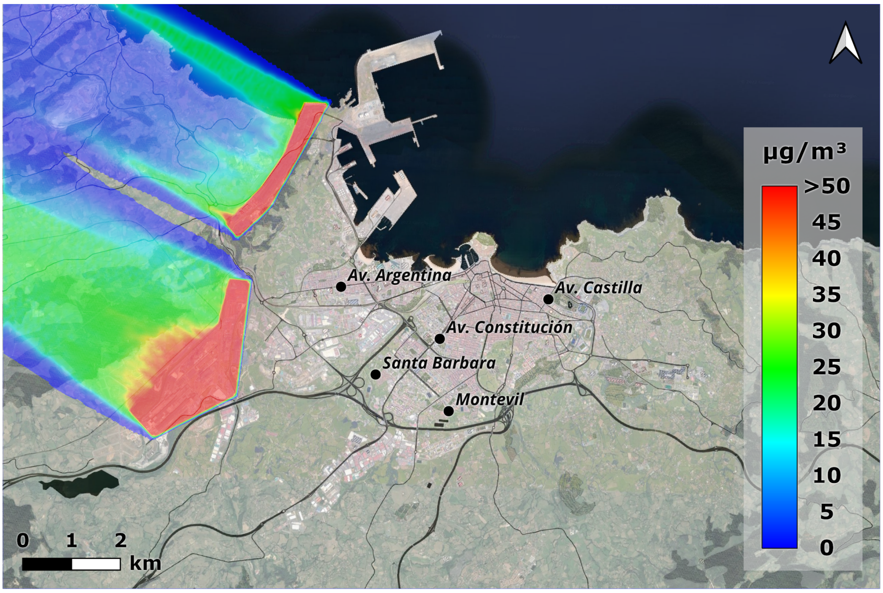


Case 5: $PM_{10}$ dispersion for East-Southeast wind (120°) at annual average speed of 2.08 m/s (ANSYS Fluent 19.2 (2019) & QGis 3.28).

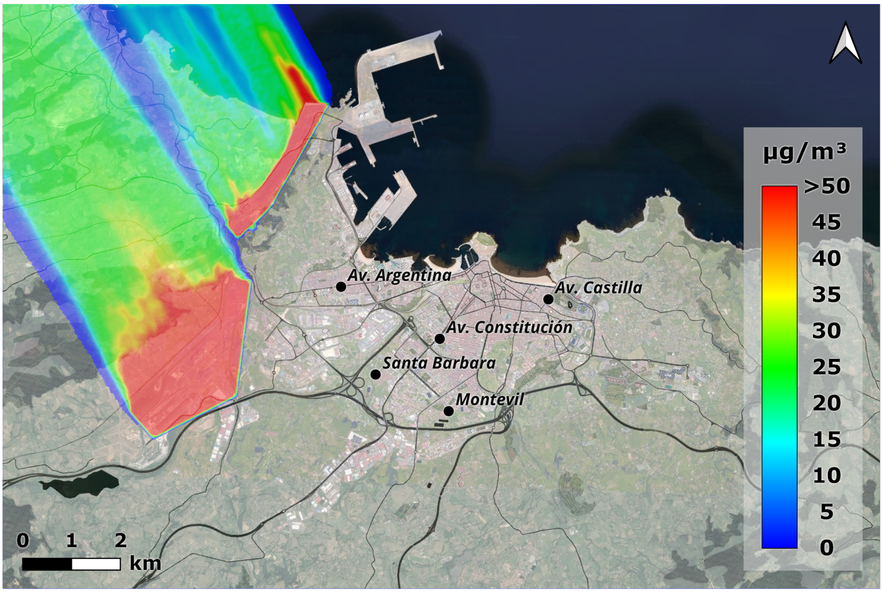


Case 6: $PM_{10}$ dispersion for South-Southeast wind (150°) at annual average speed of 1.36 m/s (ANSYS Fluent 19.2 (2019) & QGis 3.28).

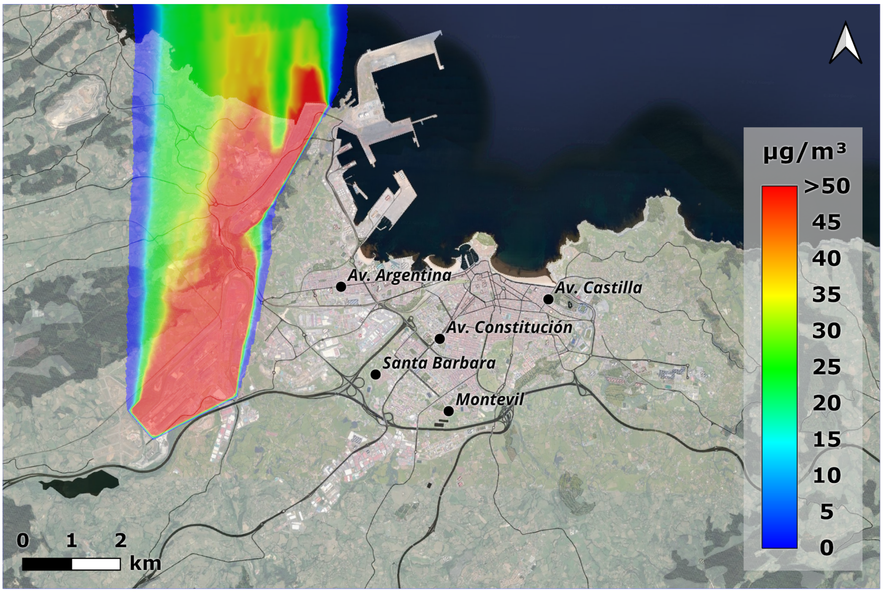


Case 7: $PM_{10}$ dispersion for South wind (180º) at annual average speed of 1.35 m/s (ANSYS Fluent 19.2 (2019) & QGis 3.28).

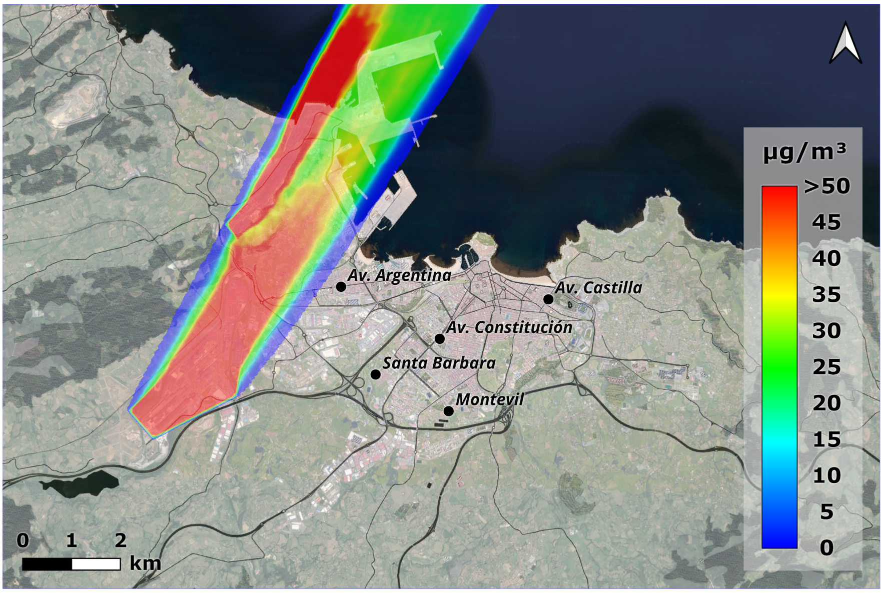


Case 8: $PM_{10}$ dispersion for South-Southwest wind (210º) at annual average speed of 1.66 m/s (ANSYS Fluent 19.2 (2019) & QGis 3.28).

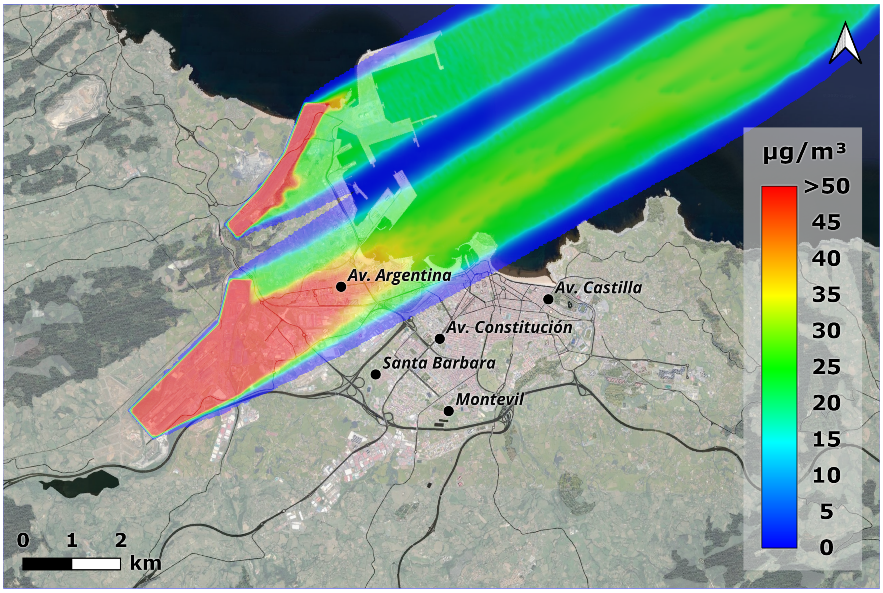


Case 9: $PM_{10}$ dispersion for West-Southwest wind (240º) at annual average speed of 1.55 m/s (ANSYS Fluent 19.2 (2019) & QGis 3.28).

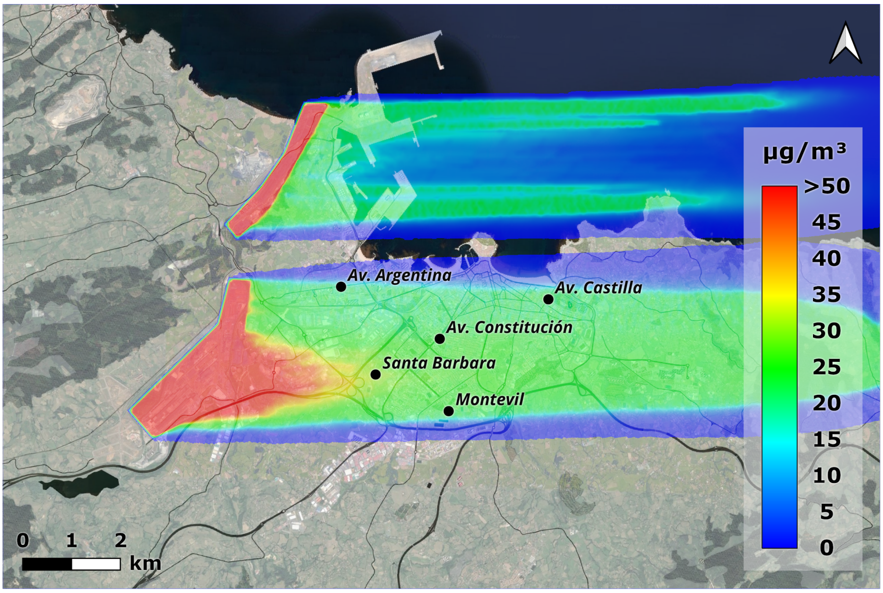


Case 10: $PM_{10}$ dispersion for West wind (270º) at annual average speed of 1.54 m/s (ANSYS Fluent 19.2 (2019) & QGis 3.28).

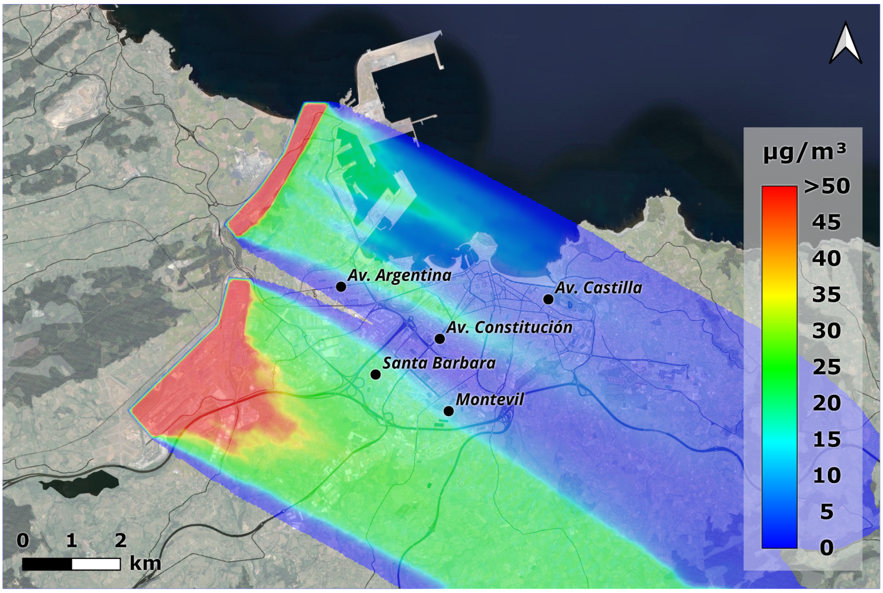


Case 11: $PM_{10}$ dispersion for West-Northwest wind (300°) at annual average speed of 1.49 m/s (ANSYS Fluent 19.2 (2019) & QGis 3.28).

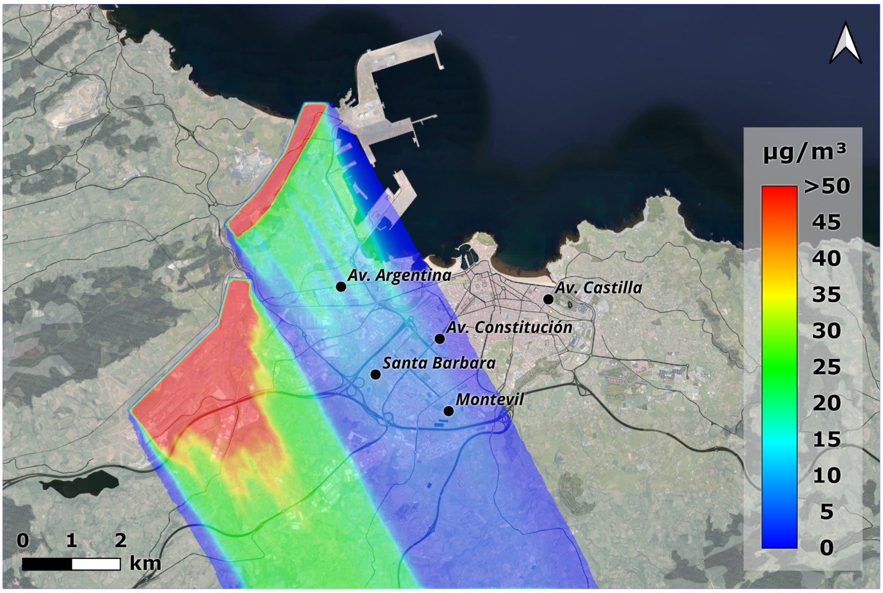


Case 12: $PM_{10}$ dispersion for North-Northwest wind (330°) at annual average speed of 1.53 m/s (ANSYS Fluent 19.2 (2019) & QGis 3.28).

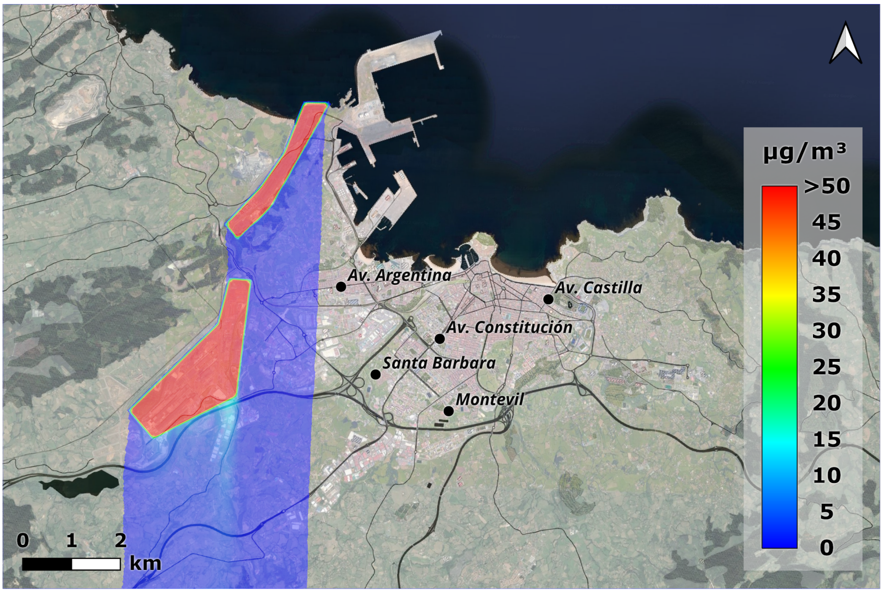


Case 13: $PM_{10}$ dispersion for North wind (0º) at annual maximum speed of 14.40 m/s (ANSYS Fluent 19.2 (2019) & QGis 3.28).

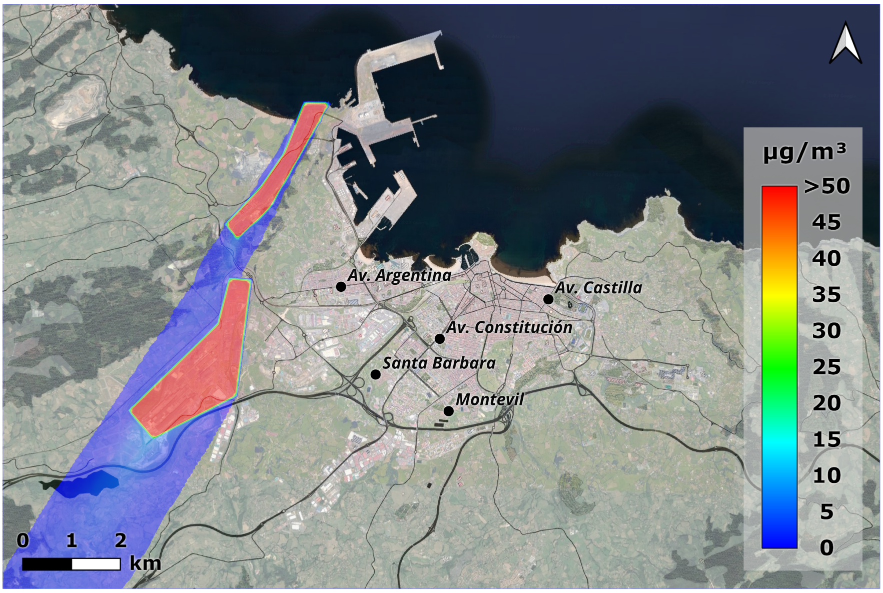


Case 14: $PM_{10}$ dispersion for North-Northeast wind (30º) at annual maximum speed of 19.90 m/s (ANSYS Fluent 19.2 (2019) & QGis 3.28).

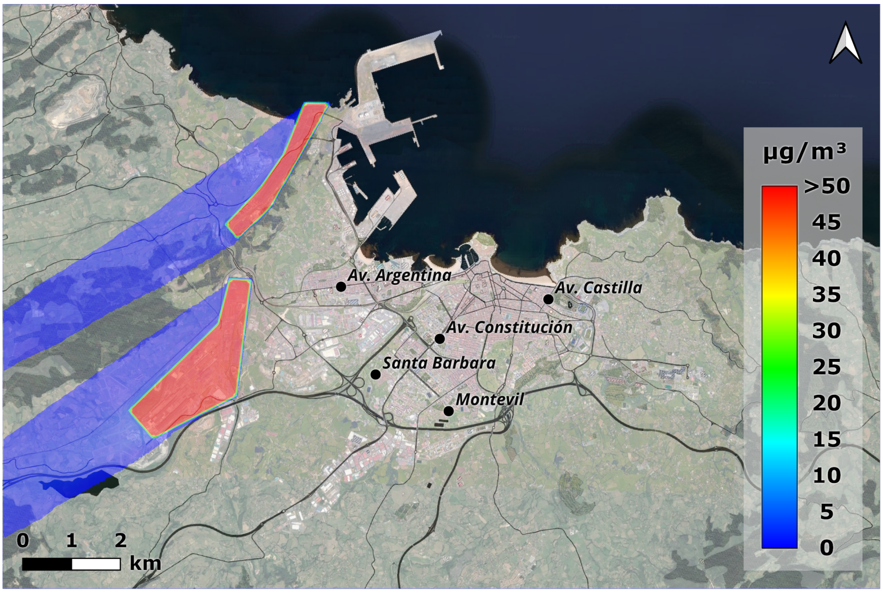


Case 15: $PM_{10}$ dispersion for East-Northeast wind (60º) at annual maximum speed of 24.30 m/s (ANSYS Fluent 19.2 (2019) & QGis 3.28).

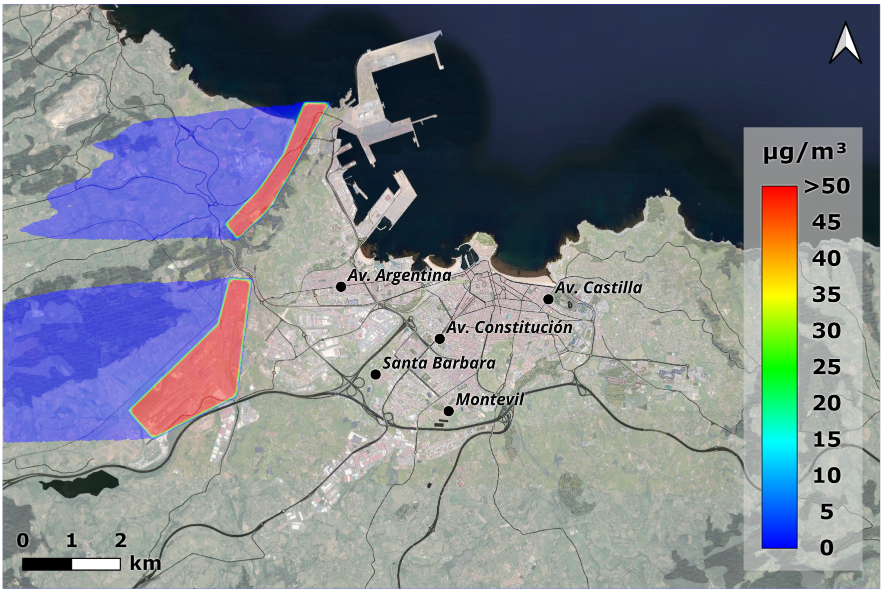


Case 16: $PM_{10}$ dispersion for East wind (90º) at annual maximum speed of 24.30 m/s (ANSYS Fluent 19.2 (2019) & QGis 3.28).

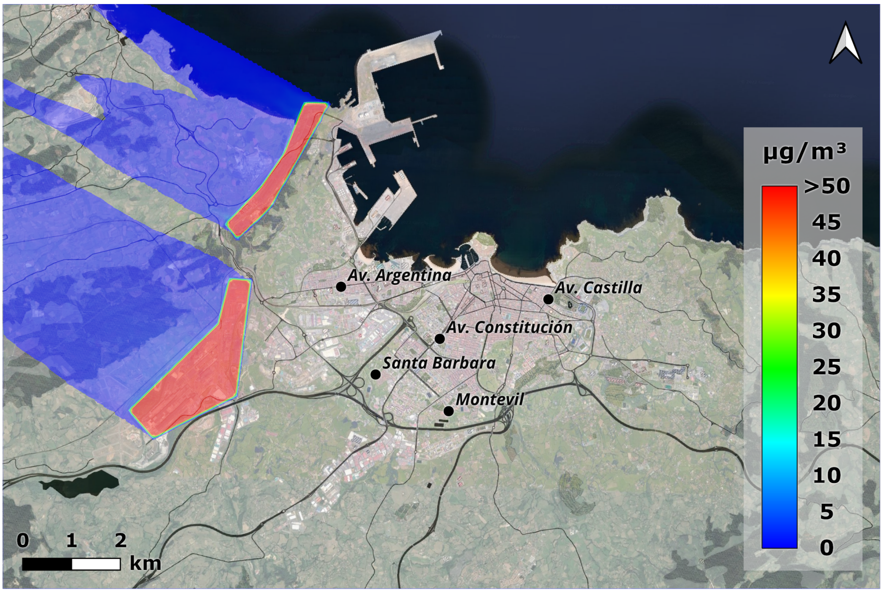


Case 17: $PM_{10}$ dispersion for East-Southeast wind (120°) at annual maximum speed of 18.90 m/s (ANSYS Fluent 19.2 (2019) & QGis 3.28).

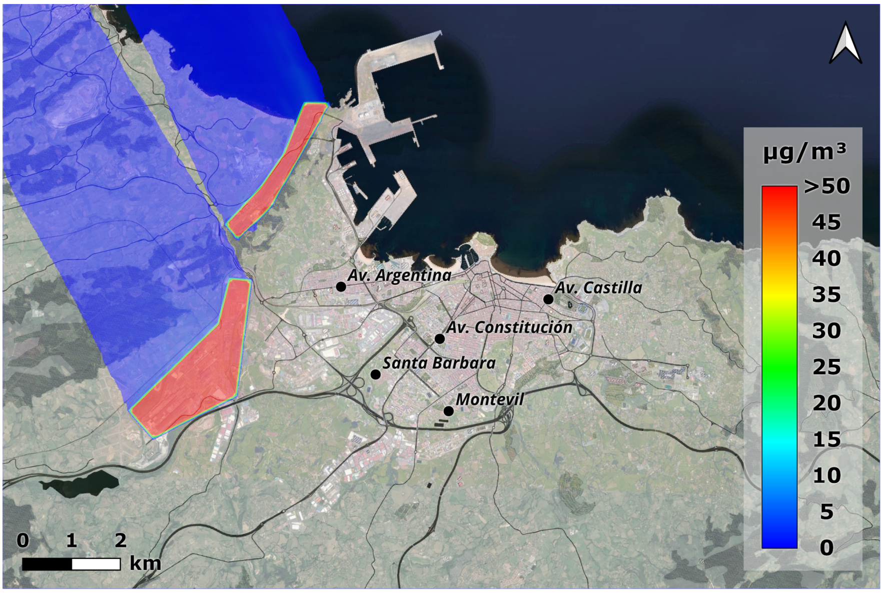


Case 18: $PM_{10}$ dispersion for South-Southeast wind (150°) at annual maximum speed of 19.10 m/s (ANSYS Fluent 19.2 (2019) & QGis 3.28).

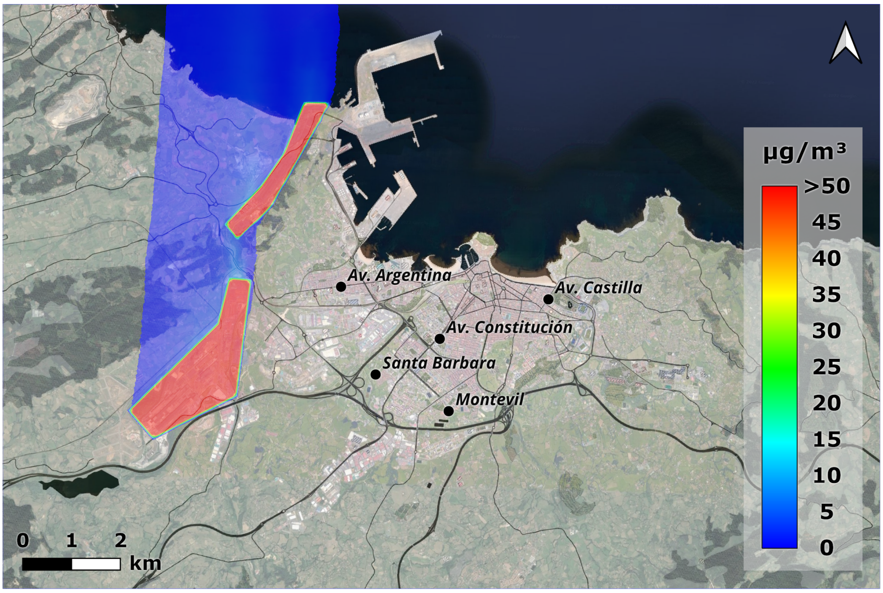


Case 19: $PM_{10}$ dispersion for South wind (180°) at annual maximum speed of 19.00 m/s (ANSYS Fluent 19.2 (2019) & QGis 3.28).

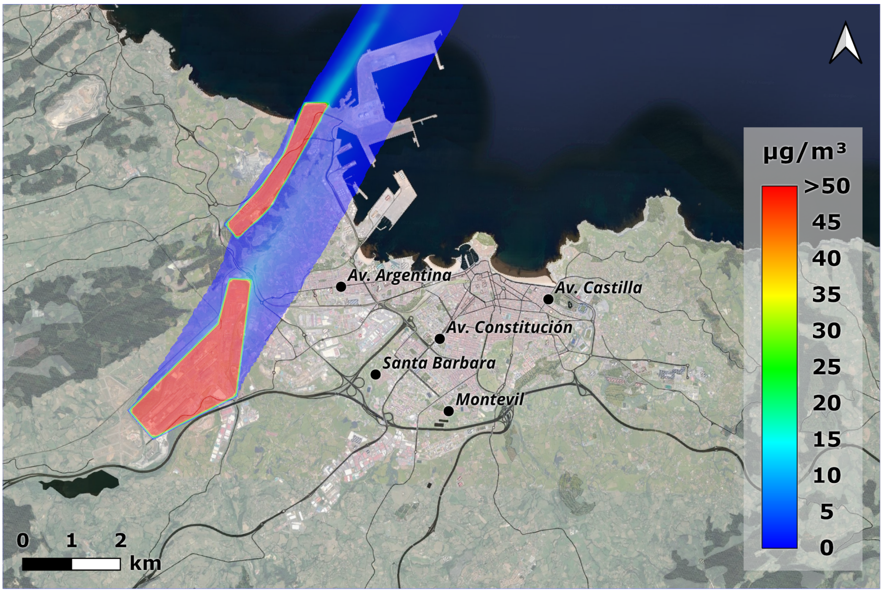


Case 20: $PM_{10}$ dispersion for South-Southwest wind (210°) at annual maximum speed of 19.20 m/s (ANSYS Fluent 19.2 (2019) & QGis 3.28).

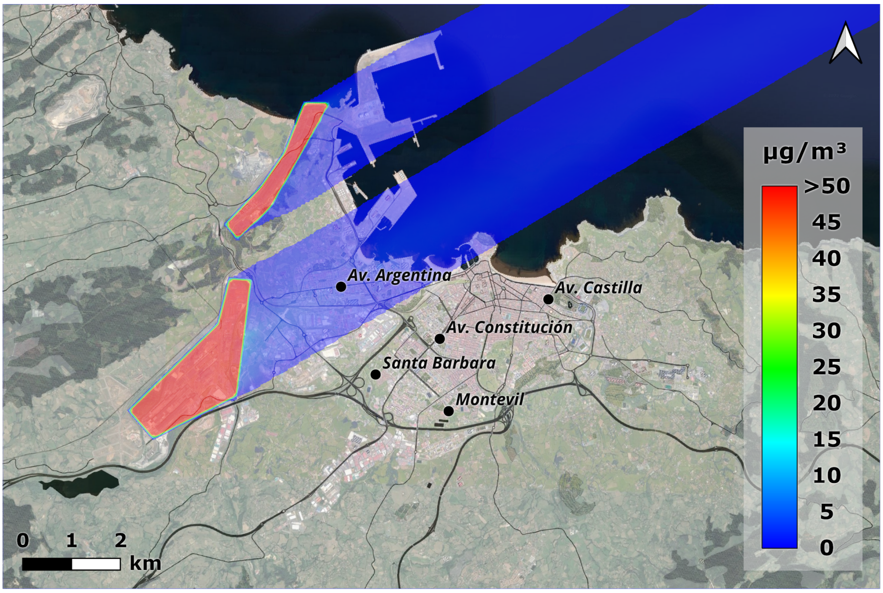


Case 21: $PM_{10}$ dispersion for West-Southwest wind (240º) at annual maximum speed of 18.00 m/s (ANSYS Fluent 19.2 (2019) & QGis 3.28).

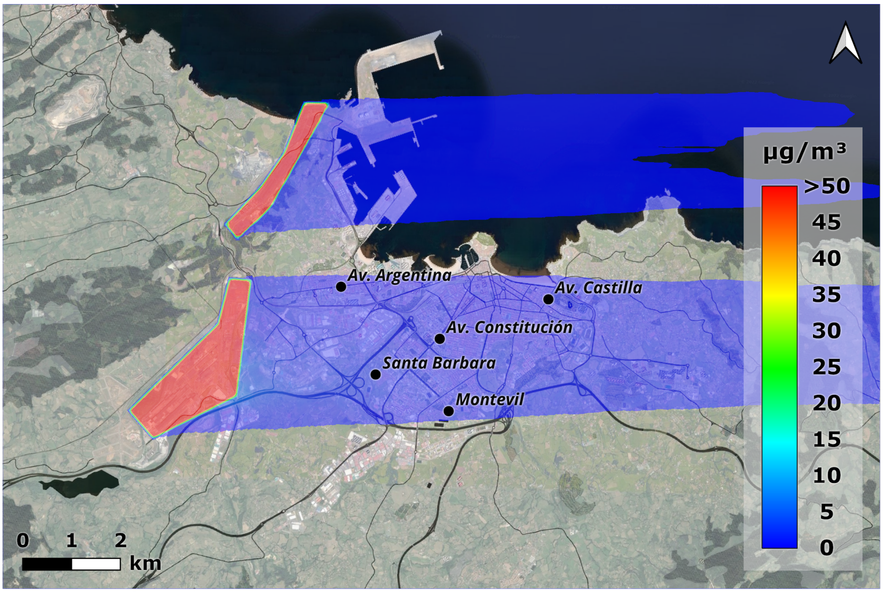


Case 22: $PM_{10}$ dispersion for West wind (270º) at annual maximum speed of 16.20 m/s (ANSYS Fluent 19.2 (2019) & QGis 3.28).

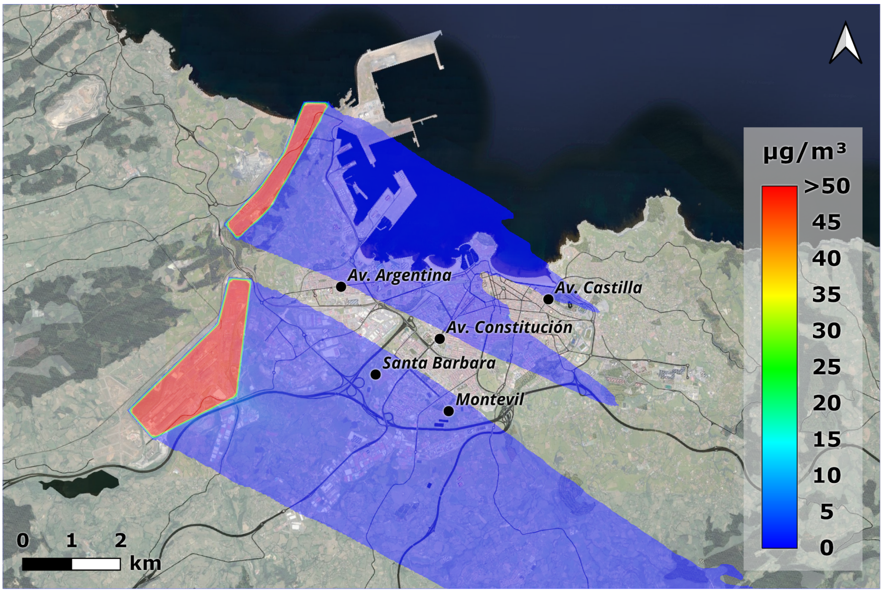


Case 23: $PM_{10}$ dispersion for West-Northwest wind (300º) at annual maximum speed of 17.10 m/s (ANSYS Fluent 19.2 (2019) & QGis 3.28).

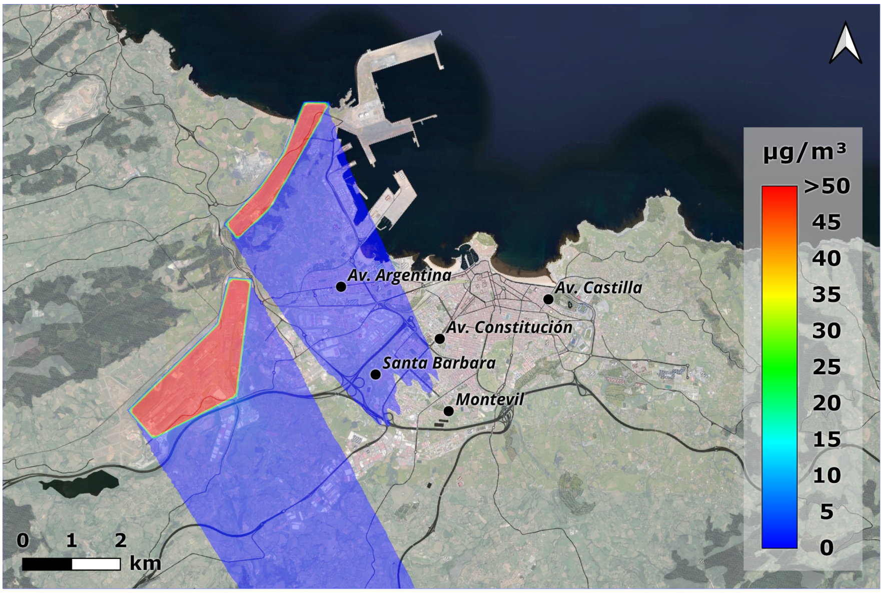


Case 24: $PM_{10}$ dispersion for North-Northwest wind (330º) at annual maximum speed of 19.80 m/s (ANSYS Fluent 19.2 (2019) & QGis 3.28).
